# Cryopreservation of Duckweed Genetic Diversity as Model for Long-Term Preservation of Aquatic Flowering Plants

**DOI:** 10.3390/plants12183302

**Published:** 2023-09-18

**Authors:** Anton Peterson, Olena Kishchenko, Markus Kuhlmann, Henning Tschiersch, Joerg Fuchs, Natalia Tikhenko, Ingo Schubert, Manuela Nagel

**Affiliations:** 1 Leibniz Institute of Plant Genetics and Crop Plant Research (IPK) OT Gatersleben (ROR (Research Organization Registry)-ID of IPK: https://ror.org/02skbsp27), Corrensstraße 3, 06466 Seeland, Germany; kishchenko@ipk-gatersleben.de (O.K.); kuhlmann@ipk-gatersleben.de (M.K.); tschiers@ipk-gatersleben.de (H.T.); fuchs@ipk-gatersleben.de (J.F.); tikhenko@ipk-gatersleben.de (N.T.); schubert@ipk-gatersleben.de (I.S.); 2 Institute of Cell Biology and Genetic Engineering, National Academy of Science of Ukraine, Acad. Zabolotnogo Str. 148, 03143 Kyiv, Ukraine

**Keywords:** duckweed, cryopreservation, vitrification, PVS3, the operating efficiency of photosystem II, *CBF/DREB1* genes

## Abstract

Vegetatively propagating aquatic angiosperms, the Lemnaceae family (duckweeds) represents valuable genetic resources for circular bioeconomics and other sustainable applications. Due to extremely fast growth and laborious cultivation of in vitro collections, duckweeds are an urgent subject for cryopreservation. We developed a robust and fast DMSO-free protocol for duckweed cryopreservation by vitrification. A single-use device was designed for sampling of duckweed fronds from donor culture, further spin-drying, and subsequent transferring to cryo-tubes with plant vitrification solution 3 (PVS3). Following cultivation in darkness and applying elevated temperatures during early regrowth stage, a specific pulsed illumination instead of a diurnal regime enabled successful regrowth after the cryopreservation of 21 accessions of *Spirodela*, *Landoltia*, *Lemna*, and *Wolffia* genera, including interspecific hybrids, auto- and allopolyploids. Genome size measurements revealed no quantitative genomic changes potentially caused by cryopreservation. The expression of *CBF/DREB1* genes, considered as key factors in the development of freezing tolerance, was studied prior to cooling but was not linked with duckweed regrowth after rewarming. Despite preserving chlorophyll fluorescence after rewarming, the rewarmed fronds demonstrated nearly zero photosynthetic activity, which did not recover. The novel protocol provides the basis for future routine application of cryostorage to duckweed germplasm collections, saving labor for in vitro cultivation and maintaining characterized reference and mutant samples.

## 1. Introduction

The Lemnaceae (duckweeds) are the smallest and fastest growing aquatic monocotyledonous flowering plants. Due to their high adaptability to environmental conditions (temperature, pH, nutrient availability, and presence of toxic compounds), high growth rate, and capability to be dispersed by migratory waterfowl, duckweeds are widely distributed on all continents except Antarctica [1]. Due to their small size, easy maintenance, and fast vegetative multiplication, as well as the availability of published data on their physiology and biochemistry, duckweeds are considered as a model system in plant biology [2]. The potential of the practical use of duckweed is very broad; it can be used for phytoremediation of polluted waters [3], for biofuel production, for poultry and livestock feeding, for human consumption [2], and as a bioreactor for recombinant proteins [4].

The fast growth of duckweed populations is ensured by vegetative propagation, while formation of flowers and seeds occurs rarely, possibly due to the fact that some species of duckweed are interspecific hybrids [5,6]. Therefore, a range of collections of wild-type duckweed clonal isolates is maintained in different laboratories [7]. Development of genetic transformation methods [8,9,10], targeted gene editing [11], and mutant induction of duckweeds [12,13] made it necessary to preserve collections of generated transgenic and mutant duckweed lines [2].

Cryopreservation is currently considered to be the most efficient method for the long-term preservation of vegetatively propagating plant germplasm and an alternative to permanent in vitro maintenance [14]. Among cryopreservation methods, vitrification is the most used approach for plant material due to its relative simplicity and applicability to large germplasm collections, and independence from specialized controlled cooling equipment [15]. Cryopreservation by vitrification requires a preliminary increase in the cellular solute concentration often achieved by dehydration and cryoprotection of tissue to enter a glassy state during fast cooling in liquid nitrogen. During vitrification, plant tissues are dehydrated by submersing them into plant vitrification solution (PVS) to prevent the risk of ice crystal formation during cooling and warming procedures. Substances with high affinity to water molecules, such as sucrose, glycerol, and/or other polyols in high concentration, are used as major components of PVSs. These substances redistribute water by osmosis and by establishing hydrogen bounds between their hydroxyl groups and H_2_O molecules [16]. Dimethyl sulfoxide (DMSO) is another widely used cryoprotectant in PVS compositions. DMSO penetrates fast in to tissue [17] and increases water permeability of bio-membranes to intensify dehydration, as well as stabilizes membranes during cooling–warming and demonstrates antioxidant properties. However, DMSO may also lead to partial DNA and RNA denaturation, and to delayed cell cycle progression, enhanced Z-form of DNA, and epigenetic alterations [18]. While the composition of PVS2 and PVS1 contain DMSO in the range of 5–15% [19], PVS3 does not contain DMSO [20] and is therefore the preferred option for the subsequent study.

So far, Lemnaceae proved recalcitrant to conventional cryopreservation techniques because living in fresh water habitats makes them sensitive to desiccation stress. In addition, the duckweed fronds contain a thick layer of aerenchyma with considerable air chambers, which provide reliable buoyancy of duckweed. The surface of the air-faced side is similar to the adaxial surface of terrestrial plant leaves with stomata, cuticle, and wax layers, while the water-faced side additionally is covered with lignin/suberin layers [21], which also may impede the penetration of PVS into the cells and affect regrowth after cryopreservation.

The first successful application of cryopreservation by vitrification to duckweed accessions of *Landoltia* and *Lemna* genera was reported by Sauter (1993) [22], applying glycerol solutions of 30–60% as a cryoprotectant. Regrowth after cryopreservation was shown for 16 out of 40 tested duckweed accessions. For *Spirodela*, *Wolffia*, and *Wolffiella* genera, no reproducible regrowth after cooling in liquid nitrogen was achieved. The US patent WO2011005502A3 [23] uncovers a method of cryopreservation for duckweeds, which requires 1–5 weeks of pre-cultivation in a nutrition medium containing a combination of different sugars with gradually decreasing temperature and illumination and using DMSO-containing PVS for cryoprotection. Another successful cryopreservation approach for duckweed accessions has been reported recently [24]. It is based on a droplet-vitrification method using PVS2 and includes the exposure of droplets of pre-treated explants and PVS2 carried on a so-called cryo-plate to liquid nitrogen before transferring to the cryo-vials. Direct contact between the plant material and liquid nitrogen could lead to contamination [25], thus giving rise to a potential risk for successful cryopreservation of germplasm collections.

The aim of this study was to develop a simple cryopreservation method for a wide range of Lemnaceae accessions. In addition, the effects of cryopreservation on the photosynthetic performance of duckweed fronds was examined. Moreover, to elucidate the importance of the integrative regulatory hub in plant responses to low temperature, the C-repeat binding factors/dehydration-responsive element binding protein (CBF/DREB1) transcription factors was investigated. CBF/DREB1s were described as the main link regulating cold- and drought-responsive genes [26,27,28]. Therefore, CBFs play a key role in mediating the tolerance to freezing [29,30]. Plants overexpressing *CBFs* enhance freezing tolerance [31,32], while knockout plants become more susceptible [33,34]. Therefore, we hypothesized that a high level of *CBF* expression might be predictive for cell viability after cryopreservation and searched for a potential correlation between *CBF* gene expression during cryopreservation and the subsequent ability of duckweeds to regrow.

## 2. Results and Discussion

### 2.1. Developing the Method

Excised shoot apical meristems are the most frequently used explants for cryopreservation by vitrification. During duckweed frond development, no cytohistological zonation and/or delimitation of a tunica and corpus occur at the frond tip, indicating that no conventional shoot apical meristem is formed [35]. Potentially, axillary buds can be used as explants for duckweed cryopreservation, but excision of the explants has been published as inducing an oxidative burst, resulting in reduced viability of the plant material after cryopreservation [36]. Therefore, whole duckweed fronds were used for cryopreservation, as already proposed [22]. Portions of fronds of *Le. gibba* 7742 were blotted on filter paper and exposed in 1 mL of PVS3. To ensure complete and reproducible penetration of PVS3 [37] and to avoid floating of the fronds on the surface of the PVS3, we designed a special single-use insert made of aluminum foil, which allows the fronds to be kept always submersed in PVS3 (Figures S1 and S2). Incubation was for 2 h at room temperature, as employed successfully for potato, garlic, mint, and shallot [38]. To improve the penetration of PVS3 into duckweed fronds, the first 15 min of PVS3 treatment was applied under vacuum [25,39], followed by an additional 1 h 45 min at normal pressure (after vacuum release).

After the first cryopreservation experiments following the protocol given in Appendix A, the green color of the rewarmed fronds bleached and gradually turned into white when exposed to light. Even without cooling, 15 min of incubation of duckweed fronds in PVS3 at room temperature led to bleaching of the fronds after washing and subsequent illumination (12 h, 50–60 μmol·m^−2^·s^−1^) in a phytochamber for 1 day. We hypothesized that rewarmed duckweed fronds may develop daughter fronds when bleaching is delayed. Therefore, early efforts were focused on finding conditions that delay bleaching.

#### 2.1.1. Attenuation of Toxicity of PVS3: Impact of Temperature and Illumination

A high concentration of glycerol in PVS3 (50% *w*/*v*) is reported to be toxic for many plant species [40]. We assumed that one of the key factors negatively affecting duckweed regrowth after cryopreservation is the combination of a high concentration of glycerol with light. Thus, we incubated duckweed fronds in PVS3 on ice (0 °C, general recommendation for reducing the toxic effect of a high cryoprotectant concentration) for 2 h in darkness. Unloading from PSV3 was also carried out on ice in a pre-cooled washing solution. All stages were performed in darkness. This allowed for a delay of bleaching by 5–6 days to be achieved, but without regrowth. Applying a specially designed attenuated illumination regime for regrowth (Figure S3) and gradually increasing the photosynthetic photon flux density (PPFD) and light period duration caused a delay of bleaching by up to two weeks after rewarming. However, still no regrowth was observed.

#### 2.1.2. Addition of Antioxidants and Other Compounds That Can Support Viability

ATP [41], casein hydrolysate, amino acids, polyvinylpyrrolidone, sodium thiosulfate, silver thiosulfate, dithiothreitol, glutathione, and different combinations of growth regulators (Table S1) were tested to search for support of viability and improvement in regrowth, however, without success. Thus, the addition of antioxidants and other compounds may improve already working protocols for cryopreservation [36] but is not a key in developing a protocol for duckweeds.

#### 2.1.3. Breakthrough Regrowth via Elevated Temperature

Cultivation under elevated temperatures (28–34 °C) is often used to rescue interspecific hybrid embryos [42]. Therefore, we checked this approach for rescuing rewarmed fronds of *Le. gibba* 7742 by incubation at 29 ± 1 °C during 3 weeks in a phytochamber under an attenuated light regime. Under these conditions, we observed the first regrown daughter frond derived from completely white mother fronds (Figure S4). The new fronds formed directly, without a callus stage. Overall, one regrowth event in one or two of three technical replicates of each biological replication was found and led to the use of 29 ± 1 °C in further experiments.

#### 2.1.4. Effect of Adaptation to Cold and High Osmotic Condition at Pre-Culture Stage on Regrowth

The most common approaches for pre-culture in plant cryopreservation are cold acclimation and sucrose pretreatment, the latter considered to be more effective for plant recovery, especially for frost-sensitive species [40,43]. We tested different conditions for pre-culture of *Le. gibba* 7742: (1) standard (12/12 h light/dark cycle at 25 °C/22 °C); (2) cold acclimation (4 °C, darkness), and (3) imitation of night frost (16/8 h light/dark cycle at 25 °C/−1 °C, respectively), with or without increased osmotic pressure (0.015 to 0.4 M sucrose) during 6 days. Overall, pre-cultivation in a medium with 0.4 M sucrose under standard growth conditions provided the best regrowth (Table S2) compared to other conditions and, thus, was used for further experiments.

#### 2.1.5. Illumination Regime Optimization for Early Regrowth

A light-sensitive *Arabidopsis* mutant was reported to tolerate a short-day regime with high light intensity better then a long day regime with low light intensity [44]. We previously observed that (1) rewarmed duckweeds incubated in darkness after rewarming stayed green for weeks without regrowth; (2) rewarmed duckweed exposed to attenuated light after 4 days of darkness displayed delayed bleaching but insufficient regrowth; and (3) rewarmed duckweed illuminated with a light regime of the standard growth condition bleached fast with no regrowth. Based on these observations, we hypothesized that substitution of attenuated illumination (Figure S3) by pulsed illumination of higher intensity increases regrowth. Therefore, a set of pulsed illumination regimes for the early regrowth stage with different durations of light impulses and dark intervals was applied to *Le. gibba 7742* (Table 1, Figure S5).

The “60/60” regime (Figure S5C) did not show differences in regrowth in comparison to the control attenuated illumination regime (Figure S3). Overall, only regimes “15/45” (Figure S5A) and “30/30” (Figure S5B) showed substantial increase in regrowth. Further, we observed two waves of appearance of new fronds: the first between days 6 and 7 after rewarming, and the second between days 9 and 11. Although more daughter fronds were generated under the “15/45” illumination regime, the duckweeds grew slower and did not proliferate “granddaughter” fronds compared with the “30/30” illumination. In addition, regrown fronds from the second wave for both illumination regimes did not form new fronds.

**Figure 1:**
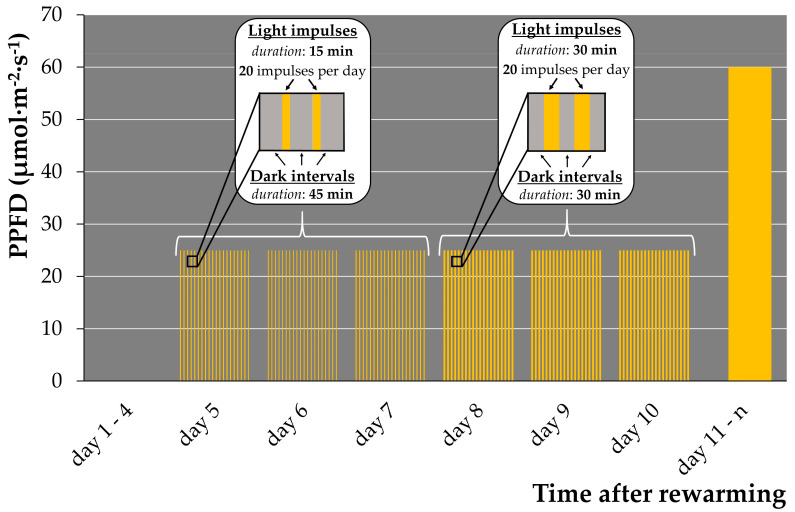
Combined pulsed illumination regime used for duckweed regrowth after cryopreservation. PPFD—photosynthetic photon flux density.

To ensure complete development and further multiplication of new generated fronds, we applied a combined pulsed illumination regime: four days of darkness, followed by three days of the pulsed “15/45” regime (best regrowth regime), and subsequently, three days under the “30/30” regime and transfer of rewarmed plants to standard cultivation condition (12/12 light/dark, 60 μmol·m^−2^·s^−1^) on day 11 (Figure 1). This pulsed illumination regime increased the regrowth of *Le. gibba* 7742 from 2–4 daughter fronds up to 9–34 per cryo-tube and ensured robust normal growth and multiplication of new fronds.

The main source of damage to dehydrated green tissues under illumination is uncoupling of carbon fixation and photosynthetic electron transport chain triggering production of reactive oxygen species and, hence, oxidative stress [45,46]. At the same time, light is an important signal for plant morphogenesis and chloroplast development [47]. Therefore, the designed pulsed illumination regime provided essential light signals for induction of duckweed frond development and, consequently, regrowth. Simultaneously, the dark intervals between light impulses were sufficient for recovery of the photosynthetic apparatus and may have suppressed a burst or enabled the scavenging of reactive oxygen species.

#### 2.1.6. Vacuum Infiltration Turned Out to Be Dispensable

Vacuum infiltration of PVS3 was used to ensure homogenous penetration of PVS3 before cooling. However, as this operation is laborious, we tested various pretreatment times with and without vacuum infiltration (Table 2) and found that the 4 h pretreatment without vacuum application produced similar results to the 4 h pre-treatment using a vacuum application. Thus, the 4 h pre-treatment in PVS3 without vacuum infiltration was used further on.

Incubation of explants in PVS on ice are recommended for many plant species [40]. The diffusion of solutions at nearly 0 °C is lower than that at room temperature; therefore, we had to increase the cryoprotection of the fronds by PVS3 up to 4 h. In addition, the saturation by cryopreservation agents of the plant tissues takes time, especially for sucrose [48]. By contrast, the cryoprotecting agent DMSO may fully penetrate leaf primordia and meristem regions within 10 min of exposure at room temperature as shown by using coherent Raman microscopy [17]. It should be noted that PVS components must not only cross the plasmalemma but also distribute throughout the whole cytoplasm volume and to desiccate cells through the redistribution of water molecules by osmosis and by establishing hydrogen bounds between their hydroxyl groups and water molecules [16]. Whatever the appropriate duration of PVS application at the cryoprotection stage is, a compromise between harmful effects of PVS and optimal desiccation of plant tissue, which is essential for successful cryopreservation, must always be found.

#### 2.1.7. Substitution of Blotting by Spin-Drying of Duckweed Fronds before Submersing in PVS3

The considerable variation in regrowth per cryo-tube under identical conditions (Table 2) indicated that one or more factors of the cryopreservation process were not reproducible. During rewarming, we frequently noticed nontransparent white layers above frozen transparent PVS3, termed “ice caps” of varying size. These ice caps are possibly the rest of the liquid nutrition medium on the surface of duckweed fronds. 

**Figure 2:**
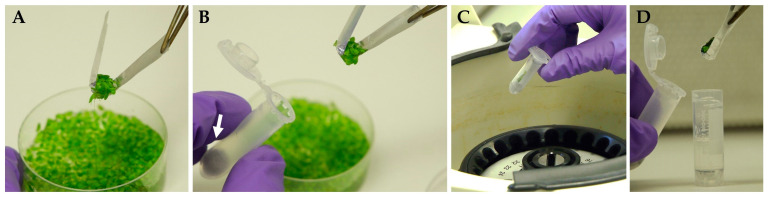
Spin-drying of duckweed fronds using a perforated aluminum insert: (**A**) collecting duckweed fronds from culture using perforated foil insert; (**B**) transferring the insert with the duckweeds into the test tube; (**C**) spin-drying by centrifugation; (**D**) transferring the insert with spin-dried duckweed from centrifugal tube into a cryo-tube filled with 1 mL of PVS3. White arrow indicates a cotton wool at the bottom of the centrifugal test tube.

To remove the surface liquid from the aquatic plant materials, spin-drying was recommended [49]. Therefore, we modified the aluminum foil insert by making a number of tiny perforations in the middle (Figure S6) and used it to collect and to transfer duckweed fronds into 2 mL U-shaped plastic centrifuge tubes (Figure 2) with a cotton wool placed on the bottom. Centrifugation at 800× *g* for 3 min allowed the liquid to drain away from fronds’ surface through the perforations onto the cotton wool.

The perforated aluminum foil was helpful for (1) duckweed sampling and transfer of the fronds to the tube, (2) spin-drying of plant material, and (3) maintaining the duckweed fronds under the PVS3 surface in cryo-tubes during cryoprotection and cryo-cooling stages. After PVS3 vitrification using a perforated aluminum foil, the regrowth results for *Le. gibba* 7742 changed from 8–31 to 16–27 per cryo-tube, i.e., were less variable, and the method was faster and less laborious. Overall, the spin-drying decreased the amount of liquid remains on the duckweed surface transferred to the cryo-tubes and eliminated ice caps after rewarming.

#### 2.1.8. Optimization of Solution Composition for Pre-Cultivation

**Figure 3:**
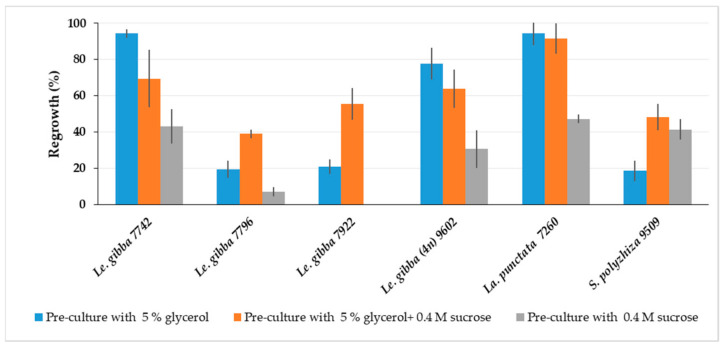
Effect of pre-culture medium on regrowth (calculated according Section 3.7.2) of different duckweed accessions at day 21 after rewarming. Error bars indicate standard deviations (n = 3).

The cryopreservation protocol developed for *Le. gibba* 7742 using 0.4 M sucrose for pre-culture was less optimal for other duckweed accessions, presumably due to a lack of adaptation to all components of PVS3. Therefore, the pre-culture medium was supplemented with either glycerol or sucrose or both for comparison and tested for *Le. gibba* (7742, 7796, 7922 and 9206), *La. punctata* 7260, and *S. polyrhiza* 9509 (Figure 3). The average regrowth of duckweed cultivated in a 24-well plate was calculated as described in Section 3.7.2. *Le. gibba* 7742 and 9206, and *La. punctata* 7260 showed the best regrowth, when only 5% glycerol was applied in the pre-culture medium. For all tested duckweed accessions, except *S. polyrhiza* 9509, regrowth after pre-culture with 0.4 M sucrose was reduced compared to that with glycerol-containing media. Combined in a pre-culture medium, 5% glycerol and 0.4 M sucrose had a positive effect on regrowth of *Le. gibba* 7796 and 7922, and *S. polyrhiza* 9509, and other tested accessions also showed quite satisfactory regrowth; thus, this variant was chosen in further experiments.

Although, the pre-culture on the medium containing both glycerol and sucrose was vital for regrowth of most tested duckweed accessions, for some of them, the pre-culture with only glycerol was preferable, probably because glycerol has a stereochemical arrangement (all the OH groups are stereochemically orientated along one side of the molecule) and small molecule size. Therefore, glycerol can interact more efficiently with the lipid bilayers and membrane proteins and can stabilize them during cryopreservation [50].

### 2.2. Regrowth of Spirodela, Landoltia, Lemna, and Wolffia Accessions after Cryopreservation

**Figure 4:**
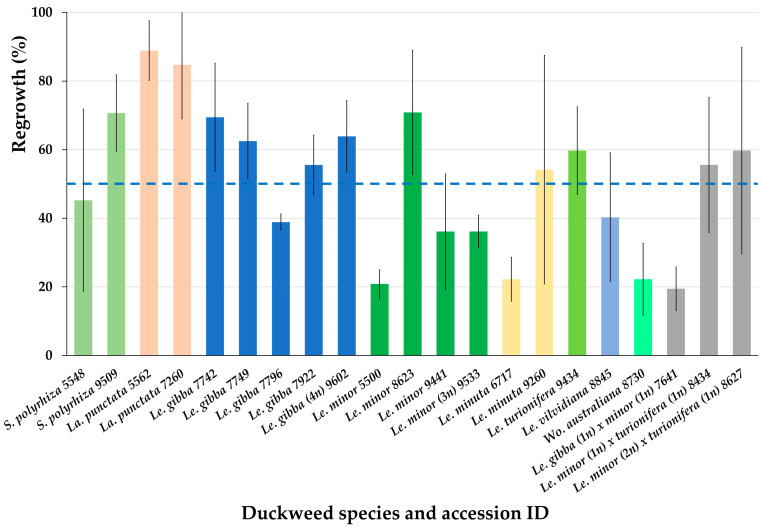
Regrowth of 21 accessions from 4 Lemnaceae genera (21 days after rewarming) obtained after applying the newly developed protocol for duckweed cryopreservation. Blue line indicates average regrowth of all tested duckweed accessions. Error bars indicate standard deviations (n = 3).

After obtaining promising regrowth results for six accession from three Lemnaceae genera (Figure 3), we cryopreserved additional 15 accessions from 4 Lemnaceae genera using the optimized protocol (Appendix B). On the average, the regrowth of all 21 cryopreserved accessions achieved 51.3 ± 20.6% (Figure 4).

Almost all tested accessions demonstrated regrowth substantially higher than 15%, the minimum viability value, established in our laboratory, and only for two of them, the regrowth was close to this cut-off value.

### 2.3. Flow Cytometry Demonstrates the Genome Size Stability after Cryopreservation

To estimate if the cryopreservation procedure has an impact on the genome size, measurements were carried out for donor cultures of autopolyploids *Le. gibba* 9206 and *Le. minor* 9533, as well as of interspecific hybrids *Le*. *gibba* × *minor* 7641, *Le. japonica* (*minor* × *turionifera*) 8434 and allopolyploid interspecific hybrid *Le. japonica* (*minor* × *turionifera*) 8627, (Table 3).

Overall, no quantitative genomic changes were found between the duckweed fronds prior to cryopreservation and the newly developed fronds after cryopreservation. Regrown duckweeds were true to type according to the data from flow cytometry; therefore, the developed method for duckweed cryopreservation could be used for long-term conservation without concern for alteration in the genome size during regrowth.

### 2.4. Monitoring the Photosynthetic Activity of Rewarmed Fronds

The observed bleaching of duckweed fronds under illumination after rewarming raised the question about a possible correlation between functional activity of the photosynthetic apparatus in chloroplasts and the regrowth of daughter fronds. To address this question, we monitored the functional activity of the photosynthetic apparatus of rewarmed fronds during two weeks after rewarming by measuring a set of chlorophyll fluorescence parameters and subsequent calculation of photosynthetic performance in samples differing in regrowth due to different pre-cultures (see Figure 3).

We measured the minimal fluorescence level (F_0_) of the dark-adapted plants, the maximal fluorescence level (F_m_) transiently induced by a saturating light pulse, the maximum fluorescence yield in the light-adapted state (F_m_′), and the fluorescence under actinic illumination (F_s_) for the *Le. gibba* 7742, *Le. gibba* 7796, *Le. gibba* 7922, and *Le. gibba* 9602 rewarmed fronds that were pre-cultured in three different solutions before cryopreservation. The photosynthetic performance was estimated by calculating the variable fluorescence (F_v_), maximum quantum yield of photosystem II (F_v_/F_m_), and operating efficiency of photosystem II (Φ_PSII_) (see Section 3.3). F_0_ derives directly from the pigment bed, primarily from the light-harvesting pigment antenna of PSII. In contrast, variable fluorescence F_v_ (difference between F_0_ and F_m_) is closely connected to the photochemical reactions. Changes in F_v_ reflect mainly the redox state of the first stable electron acceptor in PSII plastoquinone A.

**Figure 5:**
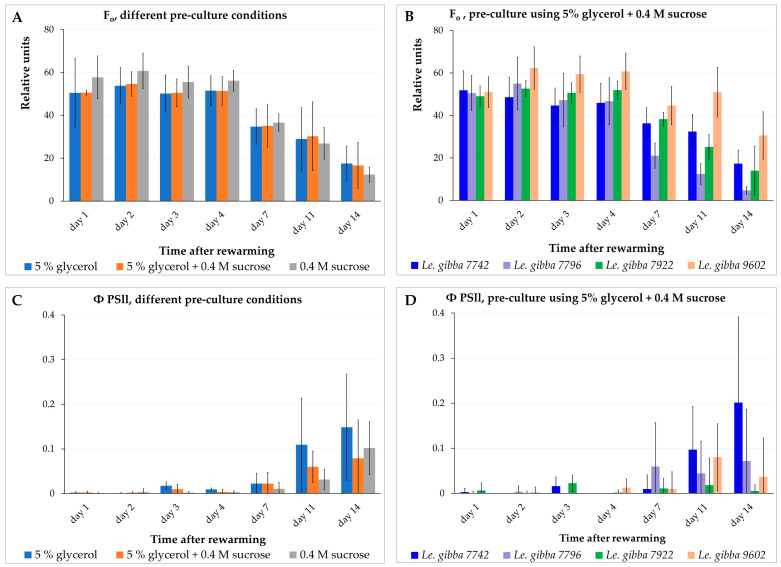
Dynamics of minimal chlorophyll fluorescence F_0_ (**A**,**B**) and operating efficiency of photosystem II Φ_PSII_ (**C**,**D**) of the rewarmed duckweed fronds depending on pre-culture (**A**,**C**) and duckweed accessions (**B**,**D**). For (**A**,**C**), data presented as average values for four accessions of *Le. gibba* (7742, 7796, 7922, 9602). Error bars indicate standard deviations (n = 3).

At the first 4 days after rewarming and cultivation in the dark, fronds displayed almost no differences in the values of F_0_ and F_m_ as well as between accessions and pre-culture conditions (Figure 5A,B and Figure S7A–C). On average, the F_0_ values for rewarmed materials were about half of that for the intact control material: 50.0 ± 10.1 (Figure 5A) vs. 90.0 ± 15.4 (Table S3). However, the F_m_ and photosynthetic performance parameters of rewarmed fronds (F_v_/F_m_ and Φ_PSII_ values) in all cases demonstrated values on average not more than 10% of values for intact control plants (Figure 5C,D and Figure S7G–L,N–Q, Table S3). These two observations suggest that, despite the substantial amount of chlorophyll in the rewarmed fronds, the photosynthetic machinery in the chloroplasts was almost nonfunctional and did not recover within two weeks after rewarming.

Between day 4 and 7 after rewarming, the F_0_ (Figure 5A,B) and F_m_ values decreased in range of 20–30% depending on accession and pre-culture solution. The observed photochemical degradation of chlorophyll was apparently the result of switching from cultivation in darkness to pulsed illumination and an elevated cultivation temperature of 29 ± 1 °C at day 5. The photosynthetic machinery did not demonstrate any signs of recovering at this period, maintaining the values of F_v_/F_m_ (Figure S5G–I,P) and Φ_PSII_ (Figure 5C,D and Figure S7J–L) at the same low level. From day 7 to 11, the F_0_ (Figure 5A,B and Figure S7A–C) and F_m_ (Figure S7N) values decreased less remarkably and even increased slightly for certain accessions and pre-culture conditions. 

From day 5 (after switching from darkness to pulsed illumination and elevated temperature), the new daughter fronds started to grow. At this stage, the surfaces of these new tiny fronds started to overlap with the surfaces of rewarmed mother fronds; thus, a precise separation between the areas of the rewarmed fronds from areas of the new fronds was impossible during fluorescence data processing. Up to day 7, the new fronds reached larger dimensions and developed their photosynthetic apparatus. Thus, subsequent decreasing of the F_0_ (Figure 5A,B and Figure S7A–C) and F_m_ (Figure S7N) values of the rewarmed fronds was partially or completely compensated by increasing of chlorophyll fluorescence of the new daughter fronds. The increasing F_v_, F_v_/F_m_, and Φ_PSII_ values at this stage for most accessions (Figure S7D–L) and pre-culture conditions (Figure S7O–Q) also point to a possible impact of newly developed fronds on the results of fluorescence measurements.

From day 11 to 14, the F_0_ and F_m_ values (Figure 5A,B and Figure S7A–C,N) decreased by half, apparently due to switching from cultivation under pulsed illumination and elevated temperature (29 ± 1 °C) to the standard condition (usual diurnal illumination and temperature 26/25 °C, 12 h/12 h light/dark period) on day 11 and further degradation of chlorophyll in rewarmed fronds and/or because a part of the tiny daughter fronds might have died or their development was interrupted. This assumption is supported by the observation that even the F_v_ values calculated for fronds that did not generate visible daughter fronds increased slightly from day 7 to 11 and decreased again after the 11th day for most of the accessions and pre-culture conditions. At this stage, differences regarding the F_0_ and F_m_ values between individual accessions appeared, suggesting that further individual optimization of switching between illumination regimes might be possible for some accessions.

The photosynthetic performance of the rewarmed fronds remained—with minor variations—at a low level during the first week of the observation (Figure 5C,D and Figure S7D–L,O–Q). This indicated that the photosynthetic apparatus of the fronds was irreversibly damaged during cryopreservation. 

The *Le. gibba* accessions displayed in this experiment substantially different regrowth after cryopreservation (Figure S7A–L) depending on the composition of the pre-culture solution (Figure 3). However, the values of the chlorophyll fluorescence and photosynthetic performance of the rewarmed fronds, averaged by values for individual accessions and grouped by the type of pre-culture solution, demonstrated no substantial differences, neither in the level nor in their dynamics during the first week after rewarming. This indicates an absence of a correlation between the photosynthetic performance of the rewarmed fronds and regrowth after cryopreservation.

In numerous studies [53,54,55,56], dismantling thylakoid membranes and plastoglobuli formation is demonstrated for plant tissue pretreated with PVS. Chlorophyll is also degraded upon dehydration stress [45,57]. Despite the apparent presence of chlorophyll in fronds right after rewarming in darkness, the photosynthetic apparatus lost its function, as we showed by monitoring the efficiency of photosystem II. Non-functioning duckweed plastids are probably not able to recover their structure and function, and the chloroplasts of regrown plants evolved from proplastids of meristem cells [58]. Duckweed regrowth occurred only via de novo development of daughter fronds from meristems located in reproductive pockets, whereas green mother fronds were not able to survive during cryopreservation procedure. Our data confirm the statement that only meristematic cells from apical dome and youngest leaf primordia can survive after cryopreservation [59].

### 2.5. Identification of Duckweed CBFs and Their Involvement in Cryopreservation

#### 2.5.1. Identification and Characterization of *CBF/DREB1* Genes in Duckweeds

DREB protein subfamily members contain a highly conserved APETALA2/ethylene-responsive element-binding factor (AP2/ERF) domain with a characteristic valine residue at position 14 that determines the binding affinity to *cis*-regulatory dehydration-responsive elements (DRE), distinguishing them from related ERF transcription factors [60,61]. Using the tBlastN program, we identified 21 putative genes from the genome of *Le. gibba,* 28 genes of *La. punctata*, and 20 *S. polyrhiza*-encoding proteins with a DREB-specific domain (Figure S8). These protein sequences, 56 DREB proteins from *Arabidopsis* and 50 DREB proteins from rice, were taken for phylogenetic analysis. The collected DREBs were clustered into four groups (I–IV, Figure S9). Since Group A1 (CBF/DREB1) has been described to be involved in cold and freezing tolerance, a focus was taken on the CBF/DREB1 representatives of the duckweed genomes. The phylogenetic reconstruction showed that three CBFs of *Le. gibba* and two each of *La. punctata* and *S. polyrhiza* clustered in Group A1 (CBF/DREB1) together with the *Arabidopsis* CBFs AtCBF1-4. The identified CBFs are highly similar and form an individual subtree. The identified proteins from *Le. gibba*, *La. punctata*, and *S. polyrhiza*, we designated as LgCBF1, LgCBF2, LgCBF3, LpCBF1, LpCBF2, SpCBF1, and SpCBF2. Besides the AP2/ERF domain, which is involved in DNA binding, the CBFs possess the LWSY-motif in the activation domain at the C-terminus and a nuclear localization signal (NLS) near the AP2/ERF domain (Figures S8 and S10). Unlike *AtCBF1-3*, which forms a self-regulatory cluster, duckweed *CBFs* are located at a large distance from each other (*LgCBF2* and *LgCBF3*) and even on different chromosomes.

To gain insight into the transcriptional regulation of *CBF/DREB1* genes, we analyzed their promoter regions for the presence of regulatory *cis*-elements. The survey revealed both E- and G-boxes in the duckweed *CBF* promoters that indicates the possibility of regulation of their expression by low temperature and light (Table S4). All promoters of duckweed *CBF* genes contained multiple elements responsive to water stress and dehydration (MYC) as well as to methyl jasmonate, abscisic acid (ABRE), and ethylene (ERE). Compared to *Arabidopsis*, the promoters of duckweed *CBF* genes have reduced numbers of *cis*-elements for the circadian rhythm of expression.

Numerous experimental data have demonstrated that the expression of *CBFs* is regulated by a circadian clock in *Arabidopsis* [62,63]. Therefore, we measured the mRNA abundance of *LgCBF1-3* in *Le. gibba* 7742 every 4 h during a day and revealed no oscillation of mRNA abundance for *LgCBF1*, whereas *LgCBF2* and *LgCBF3* showed circadian regulation with a peak at 4:00 corresponding to the Zeitgeber unit (Figure S11). The relative mRNA abundance increased about 4- and 12-fold for *LgCBF2* and *LgCBF3*, respectively, indicating also a dependency of *LgCBF2* and *LgCBF3* on the circadian clock in duckweed. However, the diurnal rhythm in duckweed was expressed weaker than was reported for *Arabidopsis* [62,63].

The mRNA abundance of *LgCBF1-3* in response to cold and osmotic stress treatment (Figure S12), usually applied during the pre-culture step, was analyzed. Exposition of *Le. gibba* 7742 to +4 °C for 3 days resulted in an increase in *LgCBF1-3* mRNA abundance: the detected relative increase was about 28-, 103- and 131-fold, respectively, compared to the control condition (+25 °C). After the addition of 0.4 M sucrose, all tested genes were induced and showed elevated transcript levels, especially for *LgCBF1*, with an expression 80-fold higher than that under the control condition (SH, 4 °C). The highest mRNA abundance was recorded for *LgCBF1* after treatment with 0.4 M sucrose at 4 °C. 

#### 2.5.2. Dynamics of *CBF* mRNA Abundance during Cryopreservation

**Figure 6:**
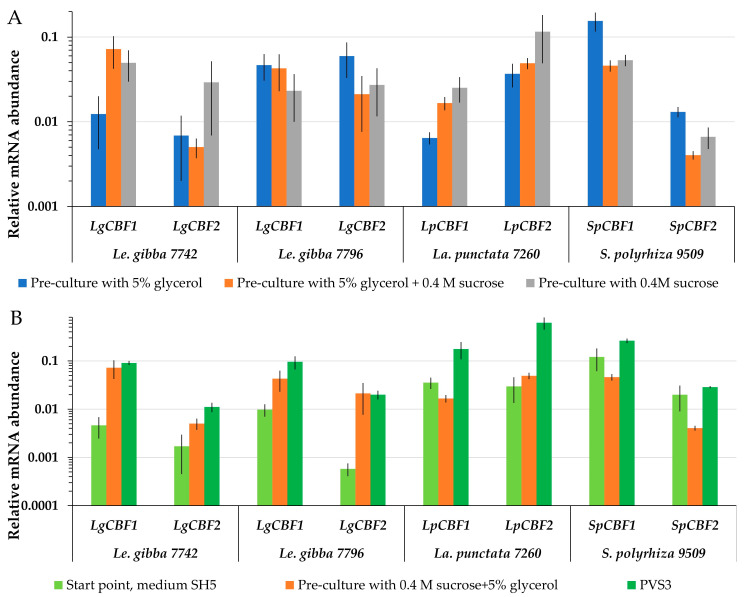
Relative mRNA abundance of duckweed *CBF1* and *CBF2* genes (**A**) after different pre-culture treatment 5% glycerol and/or 0.4 M sucrose (see Figure 3 for corresponding regrowth data); (**B**) during preparation of duckweed to cryopreservation, including PVS3 treatment. The start points were used as controls. The relative mRNA abundance was normalized against the mRNA abundance of *histone H3* (*LgH3*). Error bars indicate standard deviations (n = 5).

Searching for a potential link between the expression of *CBF* genes during cryopreservation and the ability to re-grow, the mRNA abundance of *CBF1* and *CBF2* turned out to be highly variable. The level of *LgCBF1* mRNA abundance of *Le. gibba* 7742 was the highest after pre-culture with 5% glycerol and 0.4 M sucrose (Figure 6A), and *LgCBF2* was more abundant after application of 0.4 M sucrose during pre-culture (Figure 6A). However, the highest regrowth of *Le. gibba* 7742 was observed after pre-culture of fronds with 5% glycerol (Figure 3). For *Le. gibba* 7796 and *S. polyzhiza* 9509, the highest mRNA abundance of *CBF1* and *CBF2* was recorded after pre-culture in 5% glycerol. Unlike for *Le. gibba* 7796, the regrowth efficiency of *S. polyzhiza* 9509 was higher after pre-cultivation with 5% glycerol and 0.4 M sucrose (Figure 3). The mRNA abundance of the *LpCBF1* and *LpCBF2* genes of *La. punctata* 7260 was the highest after pre-cultivation in 0.4 M sucrose, which resulted in the lowest regrowth. Thus, the mRNA abundance of *CBF* in duckweed fronds did not depend on the pre-culture conditions.

PVS3 triggered the accumulation of *CBF*. The final protocol of duckweed cryopreservation included the cultivation on SH medium followed by pre-culture in 5% glycerol plus 0.4 M sucrose and 4 h incubation in PVS3. During the pre-culture step, the *CBF1* and *CBF2* mRNA abundance was increased in *Le. gibba* 7742 and 7796 compared to the start point (Figure 6B). The following incubation in PVS3 yielded a further increase in Lg*CBF1* mRNA abundance. Transferring of *La. punctata* 7260 fronds to the pre-culture solution resulted in a slight decrease in *LpCBF1* mRNA abundance and an increase in *LpCBF2*, while mRNA abundance of both genes increased significantly after incubation in PVS3. Pre-culture of *S. polyzhiza* 9509 with 5% glycerol and 0.4 M sucrose reduced the levels of *CBF1* and *CBF2*, whereas the application of PVS3 increased them in relation to the start point. For all accessions, the regrowth after cooling and relative mRNA abundance of *CBF* genes as well as their dynamic patterns during preparation of the duckweed to cryopreservation were not correlated.

*CBFs* were reported in numerous publications to play a pivotal role in cold acclimation and acquisition of freezing tolerance [28,29,30,31,32,33,34]. In *Arabidopsis thaliana*, 12% of the cold-responsive genes are controlled by the CBF pathway, and approximately, 10% of the cold-activated genes are regulated by the HY5 pathway [64]. Thus, the CBF pathway is not the only one essential for cold response but also, in duckweed, other stress response pathways may be activated. Moreover, the expression of *CBFs* is highly regulated at many levels including transcription, mRNA stability, translation, post-translational modifications, and protein turnover. Recent studies [65] demonstrated that *Arabidopsis* CBF is activated by a redox-dependent switch. Only CBF monomers reduced by Thioredoxin h2 can induce cold-regulated gene expression, indicating the importance of the cellular redox potential in cold response. The mRNA abundance of *CBF* showed no correlation with the frequency of duckweed regrowth; thus, it cannot be used as a marker for further optimization of the cryopreservation procedure.

## 3. Materials and Methods

### 3.1. Plant Material and Growth Conditions

Duckweed accessions used in this study are from a stock collection at the Matthias Schleiden Institute—Plant Physiology, University of Jena, and were kindly provided by Klaus Appenroth (Table 4). Duckweed cultures were aseptically maintained on a liquid Schenk and Hildebrand (SH) nutrition medium, pH 5.8 (Duchefa S0225, Haarlem, NL, USA), supplemented with 5 g/L sucrose (medium SH5) in a phytochamber set at 12/12 h light/dark cycle, at a PPFD of approximately 60 μmol·m^−2^·s^−1^ with the spectrum shown in Figure S13, and at 26 °C/25 °C for the light/dark periods. For *Wolffia australiana* 8730 cultivation, a liquid SH medium, supplemented with 5 g/L sucrose, 0.5 g/L casein hydrolysate, and 0.5 g/L yeast extract (pH 5.5), was applied. Before the cryopreservation, the duration of the last subculture of donor duckweed plants was 1–2 weeks.

For cold acclimation (see Section 2.1.4) of the duckweeds during the 6-day pre-culture prior to cryopreservation, we used +4 °C in a refrigerator or a phytochamber set in the 12/12 h light/dark cycle, at a PPFD of approximately 60 μmol·m^−2^·s^−1^ with the spectrum shown in Figure S13, and at 20 °C/1 °C for the light/dark periods. 

For chlorophyll fluorescence measurement (see Section 2.4), duckweed fronds of different accessions *Le. gibba* were pre-cultured in solutions of different composition according to Section 2.1.8. For each accession and each pre-cultivation condition, 3 cryo-tubes (as 3 technical repeats) with 25–50 separated fronds or unseparated colonies in each cryo-tube were cryopreserved. Rewarmed fronds were washed in 1.2 M sucrose solution (for 1 h, on ice, in darkness) and transferred to 0.9% glucose solution in darkness at 25 °C for further washing and revitalization. The day after rewarming and washing, 8 rewarmed separated fronds or unseparated colonies from each of three cryo-tubes of the same accession and pre-cultivation condition (8 × 3 = 24 fronds or colonies) were randomly selected and transferred to plastic Petri dishes, (Ø 9 cm) on a SH solid nutrition medium, supplemented with 0.5% sucrose, 0.5% glucose, and 1% agar (pH 7.1–7.2).

For diurnal rhythm analysis of *CBF* gene expression (see Section 2.5.1), 21 flasks were inoculated with *Le. gibba* 7742 and grown in sugar-free SH medium for 4 weeks; then, the medium was replaced with a fresh one of the same composition for another 3 days. Three independent biological replicates were randomly sampled every 4 h. The first samples were collected at 8:00, when the lighting in the phytochamber started (0:00, 4:00, 8:00, 12:00, 16:00, 20:00, and 0:00 corresponding to Zeitgeber unit).

For the gene expression analysis during preparation of the duckweed for the cryopreservation procedure (see Section 2.5.2), duckweed fronds were collected (1) before immersion of the fronds to pre-culture solution (the start point), (2) at the end of pre-culture stage, and (3) at the end of exposition in PVS3 prior to cryo-cooling in liquid nitrogen. 

### 3.2. Nuclear Genome Size Measurement

Genome size measurements were performed according to [66] using a CyFlow Space flow cytometer (Sysmex-Partec GmbH, Münster, Germany). Nuclei were isolated by chopping fronds of the duckweed clones with a sharp razor blade together with young leaf tissue of *Lycopersicon esculentum* Mill. convar. *infiniens* Lehm. var. *flammatum* Lehm. ‘Stupicke Rane’ (IPK genebank accession number: LYC 418, DOI: 10.25642/IPK/GBIS/53282) as an internal reference standard using the DNA staining kit ‘CyStainR PI Absolute P’ (Sysmex-Partec GmbH, Münster, Germany). Approximately 10,000 nuclei per sample were analyzed, and at least four independent measurements per clone were performed on two independent days. The absolute DNA contents (pg/2C) were calculated based on the mean values of the G1 peak and the corresponding genome sizes (Mbp/1C) according to [67].

### 3.3. Chlorophyll Fluorescence Measurement

Chlorophyll fluorescence parameters were measured using the pulse-amplitude-modulated (PAM) technique [68,69,70] and the FluorCam device (Photon Systems Instruments, Brno, Czech Republic) installed in an automated phenotyping platform [71]. Measurements were performed for days 1, 2, 3, 4, 7, 11, and 14 after rewarming.

In the dark-adapted state, the minimal fluorescence level (F_0_) was determined by applying a weak, pulsed measuring light (PAR ≤ 0.2 µmol photons m^−2^ s^−1^) that does not drive photosynthesis, and a saturating light pulse (800 msec; PAR: 3600 µmol photons m^−2^ s^−1^) was applied to induce the transiently maximal fluorescence level (F_m_).

Variable fluorescence (F_v_) was calculated as follows:

F_v_ = F_m_ − F_0_ (1)

The maximum quantum yield of PSII is given as F_v_/F_m_.

Subsequently, induction of chlorophyll fluorescence was followed for 3 min at a light intensity of 100 µmol photons m^−2^ s^−1^. Finally, the maximum fluorescence yield in the light-adapted state (F_m_′) was measured during exposure to a saturating light flash, and the operating efficiency of PSII (Φ_PSII_) was determined from the steady state chlorophyll fluorescence under actinic illumination (F_s_) and F_m_′ according to the following equation:

Φ_PSII_ = (F_m_′ − F_s_)/F_m_ (2)

The fluorescence data were processed using FluorCam7 software (version 1.2.5.24, Photon Systems Instruments, Brno, Czech Republic) and subsequently were exported to MS Excel for further calculation and charts drawing. During fluorescent data processing for each separated frond or unseparated colony, the individual areas were selected manually with subsequent background exclusion using a cut-off value of 20. The areas corresponding to new daughter fronds which appeared after rewarming were manually (as precise as possible) excluded from the areas of rewarmed mother fronds.

The measured values of the fluorescence parameters of each individual frond or colony were averaged for each sample by all rewarmed 24 fronds or colonies in each Petri dish (that represented 8 fronds randomly taken at the second day after rewarming from 3 cryo-tubes).

### 3.4. Identification and Analysis of CBF/DREB1 Gene Family Members of Duckweeds

To access the duckweed orthologues of genes encoding transcription factors of the CBF/DREB1 family, we searched the duckweed genome sequences using as queries the reference protein sequences of AtCBF1 (At4g25490, NP_567721.1), AtCBF2 (At4gc25470, NP_567719.1), and AtCBF3 (At4g25480, NP_567720.1) from the dicotyledonous model plant *Arabidopsis thaliana* and from the monocotyledonous model plant *Ozyza sativa* OsDREB1A (Os09g35030, AAN02486.1), and OsDREB1B (Os09g35010, NP_001409784.1), OsDREB1C (Os06g03670, NP_001407946.1), downloaded from the NCBI database [72]. The tBlastN program was used to identify *DREB* gene subfamily members in genomes of *Le. gibba* 7742a (v0.5.1, id25249), *La. punctata* 5635 DWC138 (v2, id63586), and *S. polyrhiza* 9509 (voxford_v3, id51364) available on the CoGe Comparative Genome Browser [73]. To verify the search results, we analyzed all candidate sequences using InterPro [74]. The conserved motifs in CBF/DREB1 protein sequences were found using the MEME tool [75]. Multiple alignments and phylogenetic reconstructions were performed using the function “build” of ETE3 3.1.2 [76], as implemented on the GenomeNet [77]. Alignment was performed with MAFFT v6.861b with the default options [78]. The ML tree was inferred using IQ-TREE 1.5.5 ran with ModelFinder and tree reconstruction [79]. A best-fit model according to BIC was VT+F+R7. Tree branches were tested by SH-like aLRT with 1000 replicates. To address potential cis-regulatory elements in the promotor sequences of the duckweed *CBFs*, the sequences 2000 bp upstream from the start codon of the *CBF* genes were extracted from the genome database and analyzed using the PlantCARE tool [80].

### 3.5. RNA Extraction, cDNA Synthesis, and Quantitative Real-Time RT–PCR

Total RNA was extracted using RNeasy Plant Mini Kit (Qiagen GmbH, Hilden, Germany) from 100 μg of duckweed fronds, which were collected, thoroughly blotted, frozen in liquid nitrogen, and then ground using a RNeasy Plant Mini Kit (Qiagen GmbH, Hilden, Germany). The quantity of isolated RNA was estimated using a NanoDrop One C spectrophotometer (Thermo Fisher Scientific, Waltham, MA, USA). In total, 500 ng of total RNA was used as a template for synthesis of the first cDNA strand, using RevertAid H Minus First Strand cDNA Synthesis Kit, primed by oligo(dT)_18_ (Thermo Fisher Scientific, Waltham, MA, USA), in a total volume of 20 µL and diluted to 1:5 with nuclease-free water. qRT–PCR was performed in a 384-well thermocycler QuantStudio™ 6 Flex Real-Time PCR System (Applied Biosystems™, Waltham, MA, USA) using the PowerUp™ SYBR™ Green Master Mix (Thermo Fisher Scientific, Darmstadt, Germany) and gene-specific primers (listed in Table S5). Four to five identically treated biological replicates with three technical repeats were analyzed. The resulting data were analyzed using QuantStudio™ Real-Time PCR Software v1.1 (Applied Biosystems™, Waltham, MA, USA) employing corresponding *histone H3* and *β-actin* genes as reference genes (primer sequences are given in Table S5). The level of relative expression was calculated using the 2^−ΔΔCt^ method [81] and visualized using the Microsoft Excel 2016 program.

### 3.6. Measurement of Spectral Characteristic of the Light Source in the Phytochamber

The relative spectral power distribution of light from the light sources in the phytochamber, used in experiments, was measured using the HPCS300P Spectral Illuminance Sensing Module (Hangzhou Hopoo Light and Color Technology Co., Hangzhou, China). The data processing was performed using OHSP Spectral Illuminance Analyzer V 1.42 software (Hangzhou Hopoo Light and Color Technology Co., Hangzhou, China).

### 3.7. Statistical Analysis

All experimental conditions were reproduced independently a minimum of 3 times as biological replicates. Each biological replicate included 3 technical repeats as 3 cryo-tubes, the duckweeds of which were independently processed after rewarming. Using whole duckweed fronds submersed in PVS3 as the material for cryo-cooling and due to the relatively small dimensions of duckweed fronds, we did not calculate the exact number of fronds that we transferred to the cryo-tube.

#### 3.7.1. Optimization Experiments

The number of *Le. gibba* 7742 fronds transferred to the cryo-tubes during optimization experiments (25–50 fronds per cryo-tube) was calculated by visual analysis of 40 representative photo-images of the rewarmed fronds, transferred from one cryo-tube to a glass tube with liquid nutrition medium after rewarming. To accelerate and simplify the collection and processing of the results of the optimization experiments, the regrowth events per cryo-tube, observed for all 3 biological replicates and the technical repeats for each experimental condition, were visually calculated and provided as a range from absolute minimal to maximal values.

#### 3.7.2. Calculation of Average Regrowth

The average regrowth was calculated for the experiment aimed at optimizing the solution composition for pre-cultivation for all tested duckweed accessions cryopreserved using the optimized cryopreservation protocol and for experiments of *CBF1-2* expression and chlorophyll fluorescence measurements. The regrowth was calculated for 24 separated duckweed fronds (or colonies) per experimental condition, three in each well of a 24 well plate filled with SH medium and supplemented with 0.5% sucrose and 0.5% glucose (pH 7.1–7.2). The three times 8 wells represented 3 technical repeats (corresponding to 3 cryo-tubes). Each well of the plates was photo-documented for subsequent regrowth calculation. The resulting regrowth of each well can take one of four values: 0—no regrowth event detected in the well; 1—one rewarmed mother frond or unseparated colony generated (a) new frond(s); 2—two rewarmed mother fronds or unseparated colonies generated new frond(s); and 3—three rewarmed mother fronds or unseparated colonies generated new frond(s). The values for the regrowth for each of the 8 wells with the fronds from one technical repeat were summarized (representing the number of regrowth events for a given technical repeat) and were divided by 24 (the whole number of monitored rewarmed fronds from a given cryo-tube). To obtain the average regrowth, the mean values and standard deviation were calculated for all biological replicates for each used duckweed accession and/or experimental condition. All calculations were performed using MS Excel software from Microsoft Office Standard 2019. The standard deviation values were calculated using the “STDEV.S” function.

## 4. Conclusions

A simple, fast, and DMSO-free protocol for cryopreservation by vitrification of duckweed was developed. The protocol was successfully tested on a wide range of Lemnaceae accessions of the genera *Spirodela*, *Landoltia*, *Lemna*, and *Wolffia*. Cryopreservation of duckweed did not cause obvious changes in the genome size of polyploids or interspecific hybrid accessions. Using whole duckweed fronds instead of excised meristems for cryopreservation saves time and labor and allows for rapidly changing conditions for optimization. To our best knowledge, the representatives of the Lemnaceae family are the first freshwater angiosperms being successfully cryopreserved and, therefore, could be considered as the basic model for the cryopreservation of vascular freshwater aquatics.

A combination of pulsed illumination with elevated cultivation temperature for the early regrowth stage yielded for most tested duckweed accessions a substantial increase in regrowth that was in all cases higher than the minimal viability criteria of 15%. Using a newly designed single-use perforated foil insert allowed for fast and less laborious sampling of duckweed fronds from the donor culture, providing increasing protection against contamination, especially when simultaneously working with a large number of accessions, and decreasing the variability in regrowth results by spin-drying of duckweed fronds from the rests of liquid nutrition medium before submersion in PVS3.

Despite a substantial amount of chlorophyll in the rewarmed fronds, their photosynthetic machinery in the chloroplasts was not functional and did not recover. A green color of the rewarmed fronds and chlorophyll fluorescence by itself cannot be considered for viability. However, monitoring of photosynthetic performance via measurement of the chlorophyll fluorescence parameters of rewarmed fronds can serve as sensitive and non-destructive real-time tool for the detection and estimation of early regrowth of new fronds emerging from rewarmed fronds after cryopreservation. Moreover, this method can point out potentially non-optimized elements in the protocol for early regrowth.

The mRNA abundance of *CBF* during the preparation of duckweed for cryopreservation cannot be used as a genetic marker for viability and regrowth competence after cryopreservation. Probably, the CBFs are necessary for adaptation to various abiotic stress appearing during cryopreservation but are insufficient to predict successful regrowth of duckweed after cryopreservation.

Overall, our novel cryopreservation protocol (Appendix B) provides the basis for future routine cryopreservation of duckweed germplasm collections. The outcome of our optimization experiments could be useful in developing cryopreservation for other, in particular aquatic, plant species. Further testing is necessary to expand the protocol for cryopreservation to all species of the Lemnaceae family.

## Figures and Tables

**Table 1:** Parameters of the pulsed illumination regimes for early regrowth and its impact on regrowth.

Illumination Regime	Duration of Light Impulse	Duration of Dark Intervals	Number of Light Impulses per Day	PPFD, μmol·m^−2·^s^−1^	Number of Regrowth Events after Cryopreservation, per CRYO-Tube (25–50 Fronds)
“15/45”	15 min	45 min	20	25–30	9–34
“30/30”	30 min	30 min	20	25–30	3–16
“60/60”	60 min	60 min	8	25–30	1–5
Attenuated (control)					2–4

PPFD—photosynthetic photon flux density.

**Table 2:** Duration of pre-treatment of duckweed fronds in PVS3 with and without vacuum infiltration and its impact on regrowth.

Pre-Treatment Condition	Number of Regrowth Events after Cryopreservation, per Cryo-Tube (25–50 Fronds in Cryo-Tube)
V: 5 min	0
V: 15 min	0
V: 15 min + AP: 15 min	0
V: 15 min + AP: 45 min	0–1
V: 15 min + AP: 1 h 45 min	9–35
V: 15 min + AP: 2 h 45 min	7–34
V: 15 min + AP: 3 h 45 min	9–32
AP: 5 min	0
AP: 15 min	0
AP: 30 min	0
AP: 1 h	0
AP: 2 h	1–12
AP: 3 h	3–29
AP: 4 h	8–35

V—vacuum, AP—atmospheric pressure.

**Table 3:** Genome size of polyploid and hybrid duckweed accessions before cryopreservation (donor culture) and genome size of newly developed fronds of the same accessions after cryopreservation.

	Clone ID	Species	Karyotype	Genome Size (Mbp)	
				Before Cryopreservation	After Cryopreservation
1	9602	*Lemna gibba*	4 n *	1176	1120
2	7641	*Lemna gibba* × *Lemna minor*	1 n × 1 n ***	558	571
3	8434	*Lemna minor* × *Lemna turionifera*	1 n × 1 n ***	453	451
4	8627	*Lemna minor* × *Lemna turionifera*	2 n × 1 n **	635	623
5	9533	*Lemna minor*	3 n ***	600	594

*—[51], **—[52], ***—unpublished data.

**Table 4:** The list of duckweed species, polyploids, and hybrids used for cryopreservation.

	Clone ID	Genus	Species	Continent/Region	Country	State/City
1	5548	*Spirodela*	*polyrhiza*	Asia	China	Jansu, Huaian
2	9509	*Spirodela*	*polyrhiza*	Europe	Germany	Lotschen, Stadtroda 2002
3	7260	*Landoltia*	*punctata*	Australia	Australia	Victoria, Tyrendarra
4	5562	*Landoltia*	*punctata*	Asia	Israel	Kfar Hayarok, Sharon Plain
5	7742	*Lemna*	*gibba*	Europe	Italy	Sicilia
6	7749	*Lemna*	*gibba*	Europe	Belgium	Liege, Terwagne
7	7796	*Lemna*	*gibba*	Europe	Italy	Sicilia
8	7922	*Lemna*	*gibba*	South America	Argentina	Buenos Aires
9	9602	*Lemna*	*gibba* (4n)	Europe	Italy	Sicilia
10	7641	*Lemna*	*gibba* (1n) × *minor* (1n)	Asia	Israel	Hadera, Kirket Batih
11	8623	*Lemna*	*minor*	Europe	Denmark	Ijland Alborg
12	9441	*Lemna*	*minor*	Europe	Germany	Marburg St
13	5500	*Lemna*	*minor*	Europe	Ireland	Blarney, County Cork
14	8434	*Lemna*	*minor* (1n) × *turionifera* (1n)	North America	Canada	Ontario
15	8627	*Lemna*	*minor* (2n) × *turionifera* (1n)	Europe	Denmark	Sjaelland, Copenhagen, Slangerup
16	9533	*Lemna*	*minor* (3n)	Europe	Macedonia	Krusje
17	6717	*Lemna*	*minuta*	Central America	Guatemala	Chinaltenango, Chocoyos
18	9260	*Lemna*	*minuta*	Europe	Italy	Sicilia, Catania, Bot. Garden
19	9434	*Lemna*	*turionifera*	Asia	Russia	Shelekhov, region Baikal lake
20	8845	*Lemna*	*valdiviana*	South America	Brazil	Rio de Janeiro, Sao Conrado
21	8730	*Wolffia*	*australiana*	Australia	Australia	New South Wales, Singleton, Doughboy Hollow

## Data Availability

All the data generated in this work are accessible in the main text of the article and its Supplementary tables, figures, and Appendix A and Appendix B.

## Supplementary Materials

The following supporting information can be downloaded at https://www.mdpi.com/article/10.3390/plants12183302/s1, Figure S1: Preparation of the “U-shaped” aluminum foil insert; Figure S2: Preparation of duckweed frond portions for cryopreservation using the “U-shaped” aluminum foil insert; Figure S3: The scheme of the attenuated illumination regime; Figure S4: Representative image of the regrowth of a new (daughter) fronds from rewarmed (mother) fronds of *L. gibba* 7742; Figure S5: Graphical scheme of the pulsed illumination regimes; Figure S6: Preparation of perforated aluminum foil inserts; Figure S7: Dynamics of chlorophyll fluorescence parameters and photosynthetic performance of rewarmed fronds at early regrowth stage; Figure S8: Structure of identified CBF/DREB1 proteins in *Le. gibba*, *La. punctata*, and *S. polyrhiza* compared with homologues from *Arabidopsis* and rice; Figure S9: Phylogenetic tree of DREB protein subfamily members from *Arabidopsis*, rice, *Le. gibba*, *La. punctata*, and *S. polyrhiza*; Figure S10: Sequence alignment of CBF/DREB1 proteins from *Arabidopsis*, rice, *Le. gibba*, *La. punctata*, and *S. polyrhiza*; Figure S11. Relative mRNA abundance of *Le. gibba* 7742 *LgCBF1*, *LgCBF2*, and *LgCBF3* during daytime, relative to *histone H3* (*LgH3*); Figure S12. Relative mRNA abundance of *Le. gibba* 7742 *LgCBF1*, *LgCBF2*, and *LgCBF3* genes dependent on cold and osmotic treatment, normalized against mRNA abundance of *histone H3* (*LgH3*); Figure S13: Relative spectral power distribution of light from light sources in climate chamber, used in experiments; Table S1: Effect of supplemented compounds used in different solutions on cryopreservation results of *Le. gibba* 7742 after rewarming; Table S2: Regrowth of *Le. gibba* 7742 depends on pre-culture condition; Table S3: Parameters of chlorophyll fluorescence and photosynthesis performance for intact *Le. gibbba* 7742 culture; Table S4. Distribution of potential regulatory DNA cis-elements in promoter region of *CBF* genes in *A. thaliana* and *Le. gibba* 7742; Table S5: List of RT-qPCR primers used for evaluating expression of the *CBF* genes in *Le. gibba*, *La. punctata*, and *S. polyrhiza*.

## Appendix A. Preliminary Protocol for Cryopreservation Used for First Experiments with *Le. gibba* 7742

### Appendix A.1. Donor Culture Maintenance

As described in the Material and Methods section, Section 3.1.

### Appendix A.2. Pre-Culture

A portion of the donor duckweed culture were transferred onto a liquid SH nutrition medium supplemented with 100 g/L sucrose, pH 5.7–5.8, and aseptically incubated for 16–18 h (overnight) in a climate chamber set with a 12/12 h diurnal light/dark cycle, a PPFD of approximately 50–60 μmol·m^−2^·s^−1^ with the spectrum provided in Figure S13, and a 26 °C/25 °C temperature for the light/dark periods.

### Appendix A.3. PVS3 Preparation

To prepare 100 mL of PVS3, 50 g of sucrose was intensively mixed with 28 mL of the SH medium (pH 7.1–7.2) until sucrose was almost completely dissolved; 50 g of glycerol was added, and the resulting mixture was heated in a water bath at 90–95 °C with periodic shaking until complete sucrose dissolution. The clear solution was sterilized by autoclaving at 121 °C for 20 min and stored at 4 °C until usage.

### Appendix A.4. Dehydration and Cooling Procedure

1. Prepare foil strips (≈7 mm width, ≈70 mm length). Insert the foil strips (with tweezers) to cryo-tubes (2.0 mL) to obtain a “U-form” shape formed by the strip inside the cryo-tubes. Press the side parts of the “U-shaped” foil strip to the sides of the cryo-tube with tweezers (see graphic instruction on Figure S1). Transfer the pre-formed “U-shaped” foil insert to the container for autoclavation.
2. Sterilize the prepared “U-shaped” foil insert by autoclavation.
3. In the laminar cabinet, insert the autoclaved “U-shaped” foil insert into the sterile cryo-tube using sterile tweezers.
4. In the laminar cabinet, transfer a portion of the pre-cultivated duckweed fronds to sterile filter paper using sterile tweezers for brief blotting (for 1–2 min).
5. Transfer a portion of the blotted duckweed fronds (25–50 fronds) to the cryo-tube, supplemented with the “U-shaped” foil insert by inserting the fronds between “U-shape” parts of the foil insert close to the bottom of the cryo-tube.
6. Clamp the edges of the foil strip at the top of the cryo-tube with sterile tweezers to form the foil pack with the fronds inside the cryo-tube (see graphic instruction in Figure S2).
7. Add a portion of the 1 mL of PVS3 to the cryo-tube containing duckweed fronds packed in a foil insert.
8. Load the opened test tube into the vacuum chamber (exicator after aseptic treatment, placed in the laminar cabinet).
9. Apply a vacuum (as deep as possible using a common laboratory vacuum pump).
10. Incubate the test tube under vacuum for 15 min at room temperature. Release the vacuum.
11. Transfer the cryo-tube from the exicator, and cover the cryo-tube with the sterile cap.
12. Incubate for 1 h and 45 min at room temperature.
13. Transfer the cryo-tube with the duckweed to liquid nitrogen. Store the sample in liquid nitrogen for at least several days.

### Appendix A.5. Rewarming, Washing, and Unloading

1. Prepare a glass test tube (sterile, 50 mL, with foil cap) filled with 10 mL of a sterile solution of 1.2 M sucrose (washing solution).
2. Prepare the sterile 9 g/L glucose solution, buffered by 1 g/L MES, pH 7.1–7.2 (by KOH).
3. Remove the cryo-tube from the liquid nitrogen, and defrost by heating in a water bath set at 40 °C, keeping the tube submersed in the bath until the ice in the tube almost completely melts (visual observation).
4. Immediately after defrosting (almost complete ice melting), briefly treat the cryo-tube with the antiseptic solution, open the cryo-tube in the laminar cabinet, and slightly open the foil pack in the cryo-tube with sterile tweezers by separating the edges of the foil strip.
5. Immediately transfer the foil pack with duckweed from the cryo-tube to the glass test tube, fill with the 1.2 M sucrose solution (room temperature), and close the test tube using the cap.
6. Incubate for 1 h at room temperature. During this time, most of the duckweed fronds should have separated from the foil insert and should start floating on the surface of the washing solution.
7. In the laminar cabinet, remove the solution of sucrose from the test tube (leaving the duckweed fronds in the test tube) and add 10 mL of the 9 g/L glucose solution, buffered by 1 g/L MES (pH 7.1–7.2, room temperature) to the test tube.
8. Transfer the test tube to the climate chamber, set at +25 ± 1 °C, in a light-protecting box for further unloading from the rest of the components of the PVS3. Incubate for 1 day in darkness.
9. In the laminar cabinet, remove the solution of glucose from the test tube (leaving the duckweed fronds in the test tube), and add a fresh portion of 10 mL of the 9 g/L glucose solution (pH 7.1–7.2, room temperature).
10. Transfer the test tube to the climate chamber, set at +25 ± 1 °C, in a light-protecting box for further unloading and revitalizing. Incubate for 3 days in darkness.

### Appendix A.6. Early Regrowth

1. In the laminar cabinet, remove the solution of glucose from the test tube with duckweed and add 10 mL of the liquid SH nutrition medium supplemented with 5 g/L sucrose and 5 g/L glucose (pH 7.1–7.2, room temperature).
2. Transfer the test tubes to the climate chamber, set at 12/12 h diurnal light/dark cycle, PPFD approximately 50–60 μmol·m^−2^·s^−1^ with the spectrum provided in Figure S13, and 26 °C/25 °C temperature for the light/dark periods. Incubate for further observations.

## Appendix B. Optimized Protocol for Cryopreservation of Lemnaceae Representatives

### Appendix B.1. Donor Culture Maintenance

As described in the Material and Methods section, Section 3.1. The duration of the last subculture of the donor duckweed plants should be in the range of 1–2 weeks prior to transferring to the pre-cultivation stage.

### Appendix B.2. Pre-Culture

A portion of the donor duckweed culture was transferred onto the liquid SH nutrition medium supplemented with 0.4 M sucrose and 5% glycerol (*v*/*v*), pH 5.7–5.8, and aseptically incubated for 36–48 h in a climate chamber, set at 12/12 h diurnal light/dark cycle, PPFD approximately 50–60 μmol·m^−2^·s^−1^ with the spectrum provided in Figure S13, and 26 °C/25 °C temperature for the light/dark periods.

### Appendix B.3. PVS3 Preparation

To prepare 100 mL of PVS3, 50 g of sucrose was intensively mixed with 28 mL of the SH medium (pH 7.1–7.2) until the sucrose was almost completely dissolved; 50 g of glycerol was added and the resulting mixture was heated in a water bath at 90–95 °C with periodic shaking until complete sucrose dissolution. The clear solution was sterilized by autoclaving at 121 °C for 20 min and stored at 4 °C until usage.

### Appendix B.4. Dehydration and Cooling Procedure

1. Prepare the foil strips (≈7 mm width, ≈70 mm length). Make 15–20 perforations in the central part of the foil strip using a needle or the sharp and thin end if small tweezers (to accelerate and simplify this operation, we made a special many-needled tool for this purpose from a comb for animals). Insert the foil strips into the plastic centrifugal test tube (2.0 mL) to obtain the “U-shape” form of the strip inside the test tube. Insert a magnetic stirrer bar (7 mm) or another stick with the same diameter and U-shaped end into the test tube to press the foil strip onto the internal surface of the centrifugal test tube (see graphic instruction on Figure S6). Transfer the pre-formed foil insert to the container for autoclavation.
2. Sterilize the prepared foil inserts by autoclavation.
3. Sterilize swabs by autoclavation.
4. In the laminar cabinet, transfer 1 mL of sterile PVS3 solution to the sterile cryo-tubes (2 mL). Close, and precool in ice.
5. In the laminar cabinet, insert the autoclaved swabs into the sterile plastic centrifugal test tubes (2 mL) using sterile tweezers. Push the swabs down to the bottom of the centrifugal test tubes.
6. In the laminar cabinet, transfer the portions of duckweed fronds from donor cultures (after the pre-cultivation stage) to the sterile centrifugal test tubes, supplemented with cotton wool, using autoclaved foil inserts (as single-use sampler) and sterile tweezers (see graphic instruction on Figure 2).
7. Clamp the edges of the foil strips at the top of the centrifugal test tubes with sterile tweezers to form the foil packs with the fronds inside the centrifugal test tubes.
8. Close the test tubes, and spin-dry the duckweed fronds by centrifugation for 3 min at 800× *g* at room temperature.
9. In the laminar cabinet, transfer the foil packs with spin-dried duckweed fronds to the precooled-in-ice cryo-tubes filled with 1 mL of PVS3.
10. Immediately close the cryo-tubes, and transfer it on ice in darkness.
11. Incubate for 4 h on ice in darkness.
12. Transfer the cryo-tubes with the duckweed to liquid nitrogen. Perform the operation under weak daylight (or dusk) or weak artificial light. Store the sample in liquid nitrogen for at least several days.

### Appendix B.5. Rewarming, Washing, and Unloading

1. Prepare a glass test tube (sterile, 50 mL, with foil cap) filled with 10 mL of a sterile 1.2 M sucrose solution (washing solution). Precool and keep on ice.
2. Prepare the sterile 9 g/L glucose solution, buffered by 1 g/L MES, pH 7.1–7.2 (by KOH). Precool at ≈10 °C.
3. Remove the cryo-tube from liquid nitrogen, and immediately defrost by heating in a water bath set at 40 °C, keeping the tube submersed in the bath until some signs of viscous fluidity of PVS3 in the cryo-tube appears (visual observation). Perform the operations under weak daylight (or dusk) or weak artificial light.
4. Immediately after defrosting, briefly treat the cryo-tube with the antiseptic solution, open the cryo-tube in the laminar cabinet, and slightly open the foil pack in the cryo-tube with sterile tweezers by separating the edges of the foil strip. Perform the operations under weak daylight (or dusk) or weak artificial light.
5. Immediately transfer the foil pack with duckweed from the cryo-tube to the precooled-in-ice glass test tube, fill with the 1.2 M sucrose solution, and close the test tube using the cap. Perform the operations under weak daylight (or dusk) or weak artificial light.
6. Immediately transfer the glass test tube with rewarmed duckweed on ice in darkness.
7. Incubate on ice in darkness for 1 h. During this time, most of the duckweed fronds should have separated from the surface of the foil pack and should start floating on the surface of the washing solution.
8. In the laminar cabinet, remove the solution of sucrose from the test tube (leaving the duckweed fronds in the test tube), and add 10 mL of the 9 g/L glucose solution, buffered by 1g/L MES (pH 7.1–7.2, precooled at ≈10 °C) to the glass test tube with rewarmed duckweed. Perform the operations under weak daylight (or dusk) or weak artificial light.
9. Transfer the test tube to the climate chamber, set at +25 ± 1 °C, in a light-protecting box for further unloading from the rest of the components of the PVS3. Incubate for 1 day in darkness.
10. In the laminar cabinet, remove the solution of glucose from the test tube (leaving the duckweed fronds in the test tube), and add a fresh portion of 10 mL of the 9 g/L glucose solution, buffered by 1 g/L MES (pH 7.1–7.2, room temperature). Perform the operations under weak daylight (or dusk) or weak artificial light.
11. Transfer the test tube to the climate chamber, set at +25 ± 1 °C, in a light-protecting box for further unloading and revitalizing. Incubate for 3 days in darkness.

### Appendix B.6. Early Regrowth

1. In the laminar cabinet, remove the solution of glucose from the test tube with duckweed and add 10 mL of the liquid SH nutrition medium supplemented with 5 g/L sucrose and 5 g/L glucose (pH 7.1–7.2). For *Wolffia australiana* 8730, the liquid SH medium, supplemented with 5 g/L sucrose, 5 g/L glucose, 0.5 g/L casein hydrolysate, and 0.5 g/L yeast extract (pH 7.1–7.2), should be added. Perform the operations under weak daylight (or dusk) or weak artificial light.
2. Transfer the test tubes to the climate chamber, set at +29 ± 1 °C, for re-growth. Incubate in accordance with the pulsed illumination regime (see Figure 1) with the light spectrum provided in Figure S13 and at a temperature of +29 ± 1 °C for 6 days.
3. Transfer the test tubes to the climate chamber, set at a 12/12 h diurnal light/dark cycle, PPFD approximately 50–60 μmol·m^−2^·s^−1^ with the spectrum provided in Figure S13, and 26 °C/25 °C temperature for the light/dark periods for further re-growth.
