# Supplementary material for: Cryopreservation of Duckweed Genetic Diversity as Model for Long-Term Preservation of Aquatic Flowering Plants
Source: Plants (Basel). 2023 Sep 18;12(18):3302. doi: 10.3390/plants12183302 (PMC10534739; doi:10.3390/plants12183302)
Supplement: Supplementary file 1 [file plants-12-03302-s001.zip › Peterson_et_al_Cryopreservation_of_duckweed_Suppl_Fig.pdf]

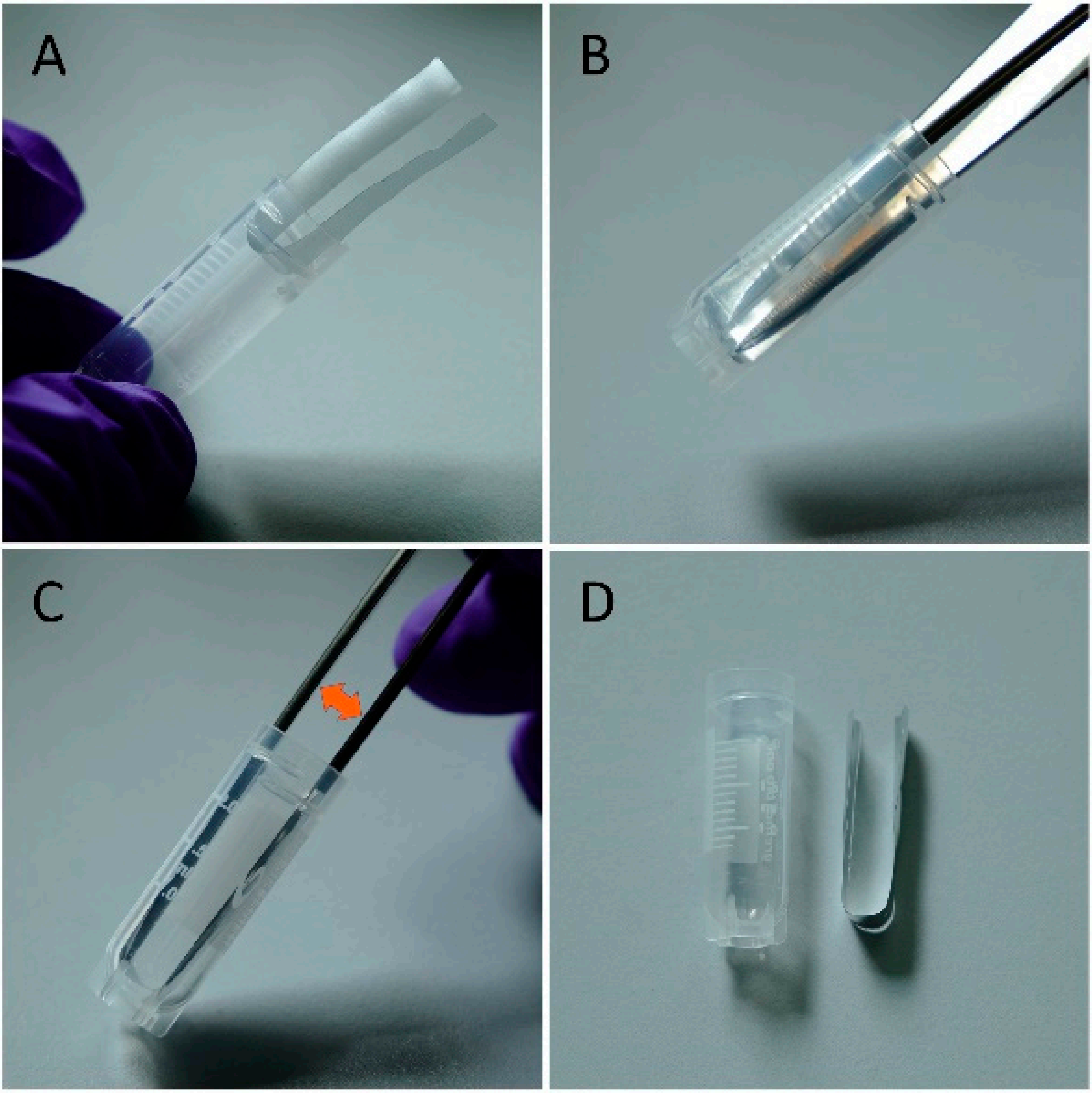

Figure S1. Preparation of the „U- shaped" aluminum foil insert: **(A)** Start of insertion of the foil strip to the cryo-tube for subsequent pre-forming; **(B)** Pushing of the strip to the bottom of the cryo-tube by tweezers; **(C)** Pre-forming the „U- shaped" insert by pressing the side parts of the „U- shaped" foil strip to the sides of the cryo-tube with tweezers, directions of pressing are shown by red arrow; **(D)** Ready to use pre-formed insert.

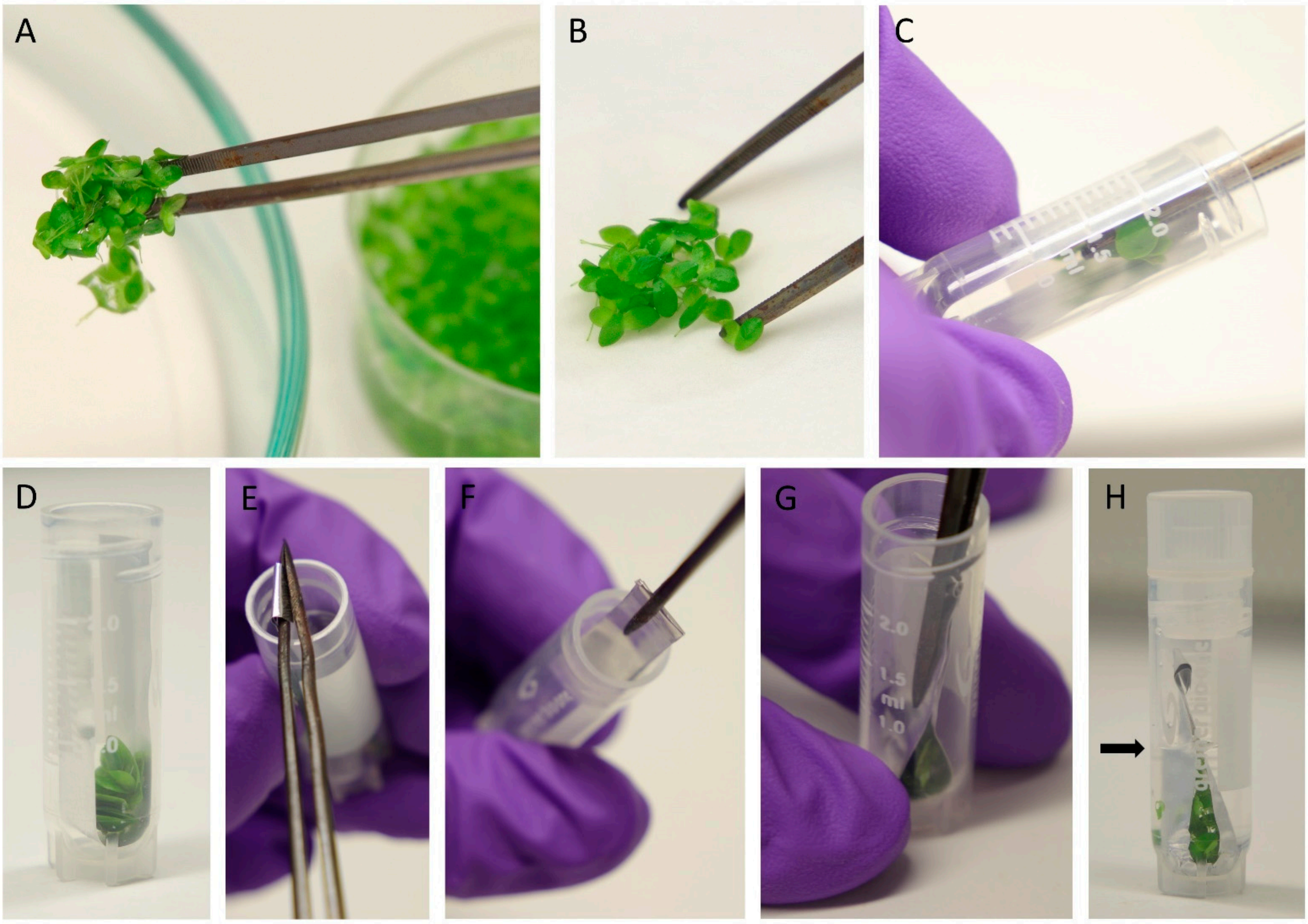

Figure S2. Preparation of duckweed frond portions for cryopreservation using the „U- shaped" aluminum foil insert: **(A)** Transferring a portion of pre-cultured duckweed fronds from a Petri dish to sterile filter paper; **(B)** Blotting the portion of the duckweed fronds on the filter paper; **(C)** Transferring the portion of blotted duckweed fronds to the cryo-tube with the „U- shaped" insert; **(D)** Placing them at the bottom of the „U- shaped" insert in the cryo-tube; **(E)** Twisting of the top parts of the aluminum foil edges of the insert; **(F, G)** Clamping the edges of the foil strip at the top of the cryo-tube with sterile tweezers to form the foil pack with the fronds inside the cryo-tube; **(H)** Ready for transferring to liquid nitrogen of the cryo-tube with portion of the duckweed fronds packed in the insert and submerged in PVS3. The portion of the duckweed fronds is retained under PVS3 level by the insert. Black arrow indicates the level of PVS3 in the cryo-tube.

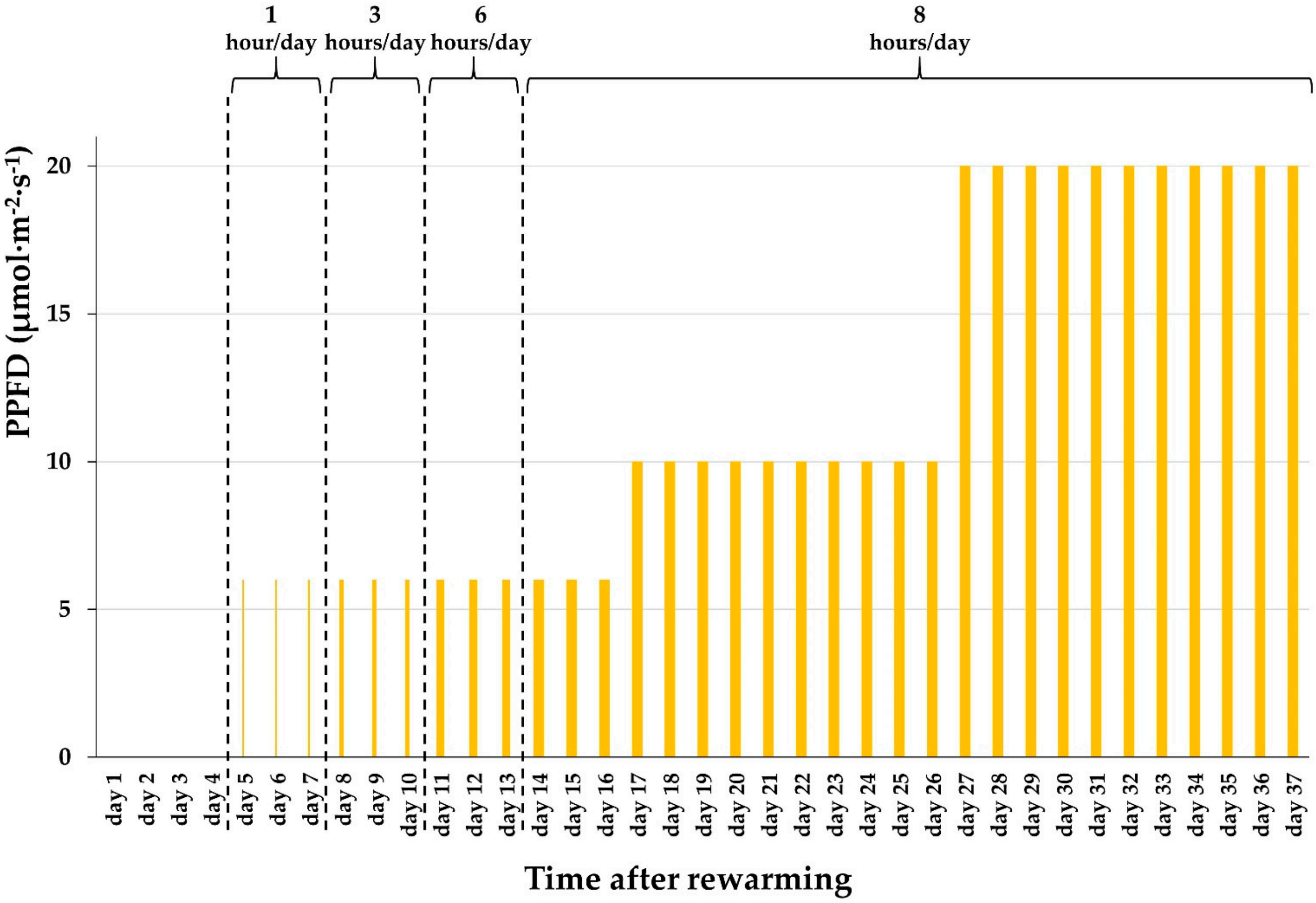

Figure S3. The scheme of attenuated illumination regime. PPFD - the photosynthetic photon flux density.

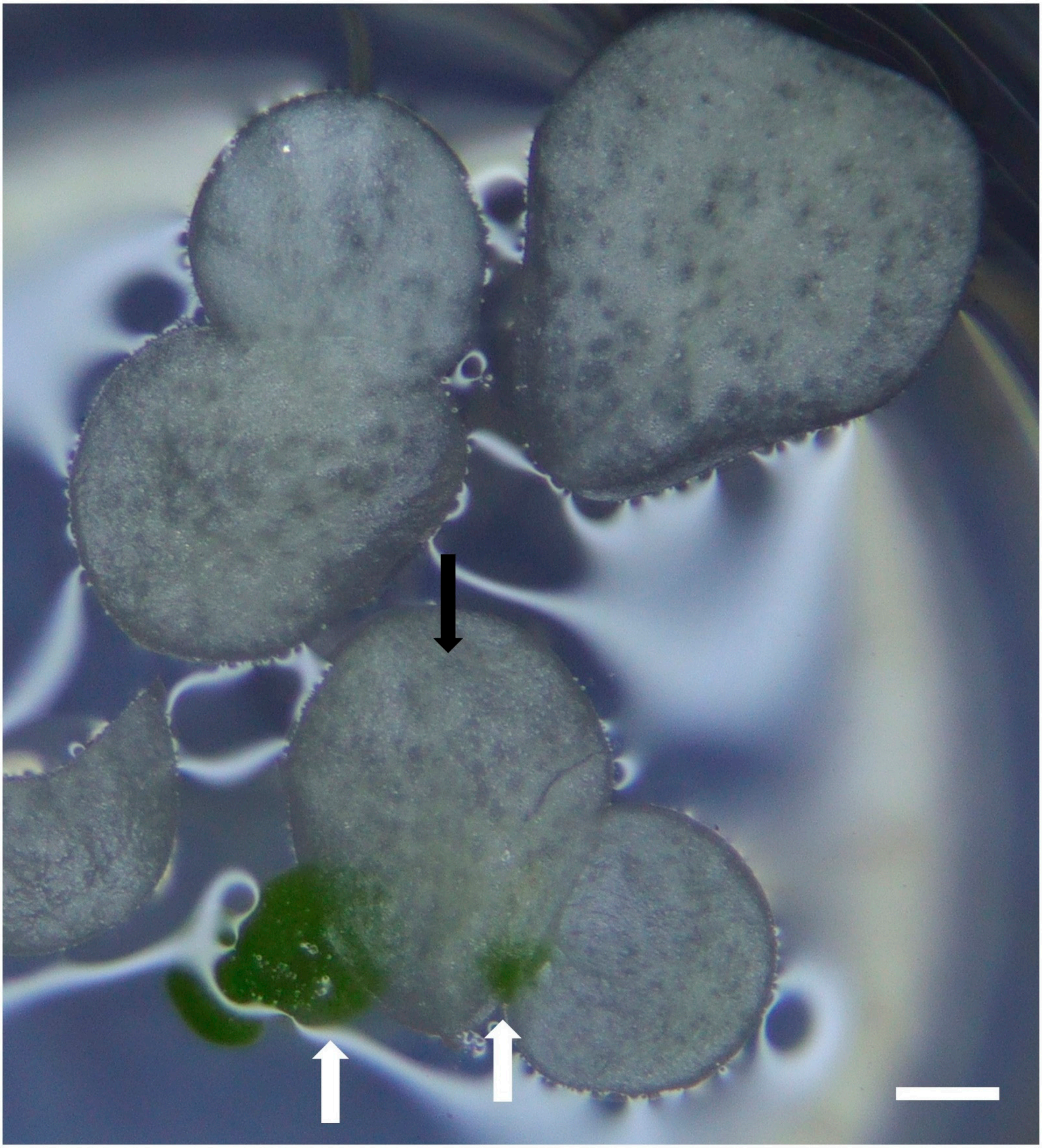

Figure S4. Representative image of the regrowth of a new (daughter) fronds from rewarmed (mother) fronds of *L. gibba* 7742. Mother fronds were already almost completely bleached at the moment of regrowth event detection. Black arrow indicates the completely bleached rewarmed (mother) frond colony. White arrows indicate the new (daughter) fronds, which appeared after rewarming. Bar correspondent to 1 mm.

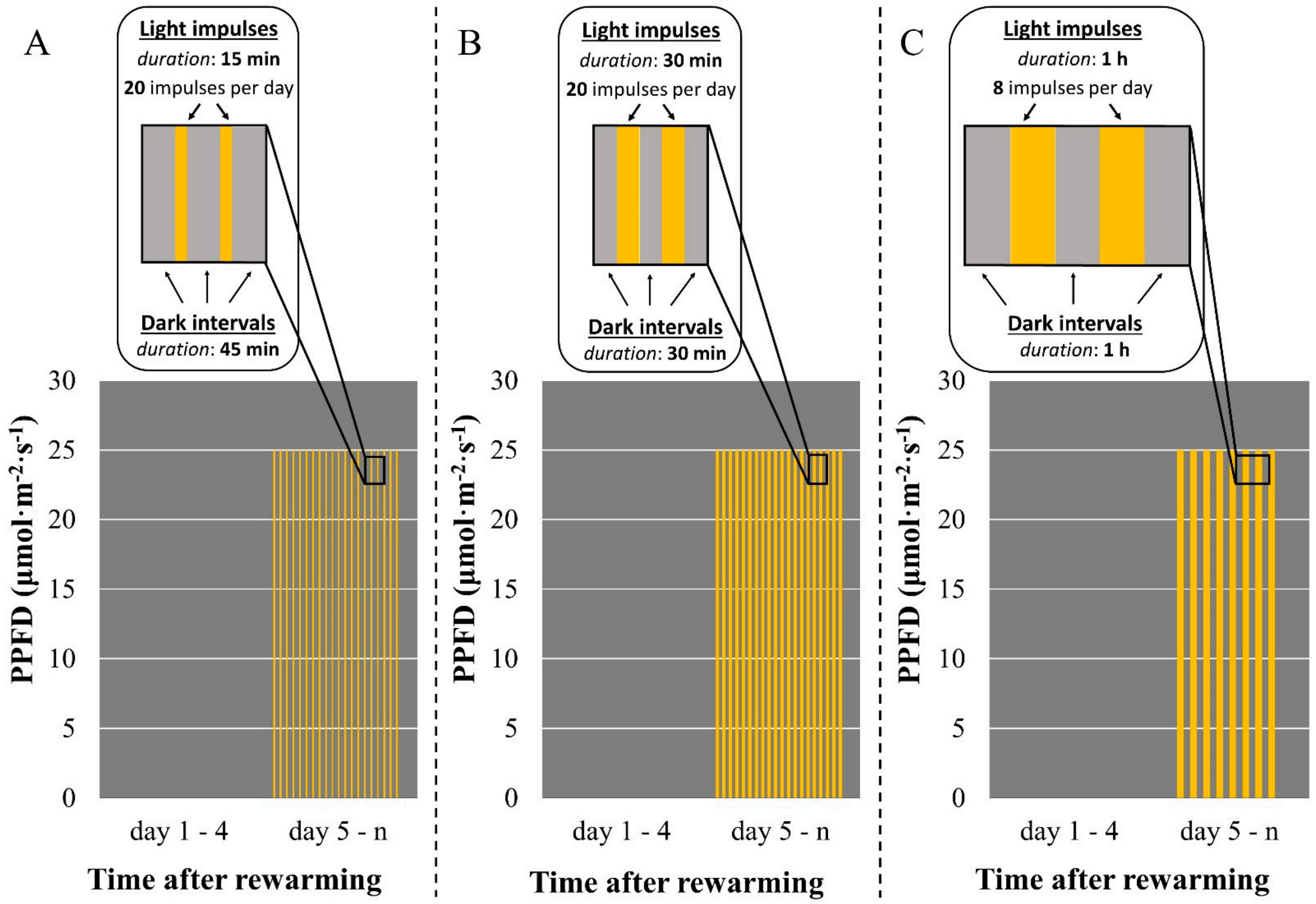

Figure S5. Graphical scheme of the pulsed illumination regimes: **(A)** "15/45"; **(B)** "30/30"; **(C)** "60/60". PPFD - the photosynthetic photon flux density.

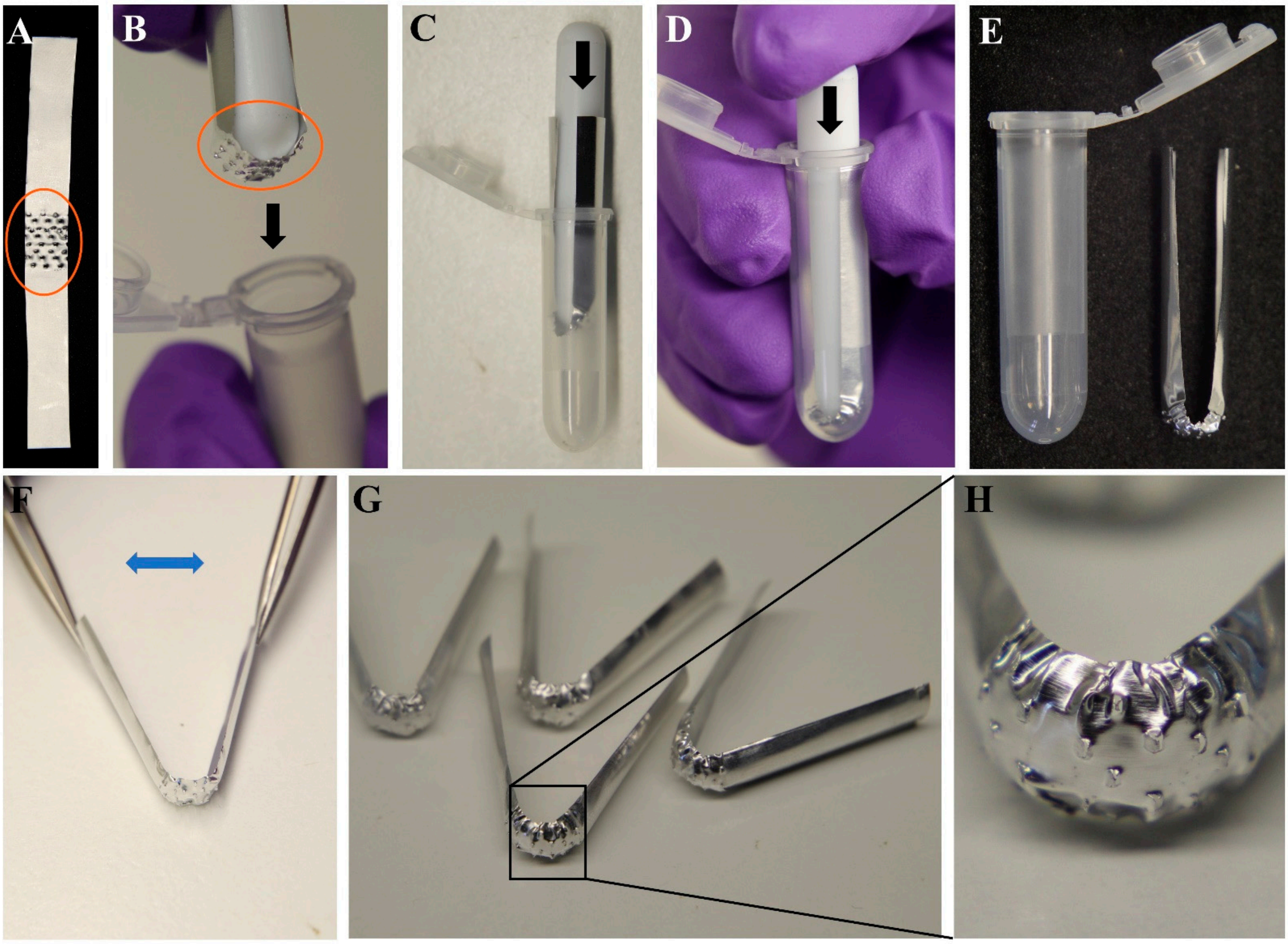

Figure S6. Preparation of perforated aluminum foil inserts: **(A)** The foil strip with perforations in the middle zone (marked by orange oval); **(B)** Insertion of the perforated foil strip to the 2 ml centrifugal test-tube using magnetic stirrer bar (8 mm diameter). Black arrow points on direction of insertion. Red oval indicates position of perforated part of the strip; **(C)** Partially inserted foil strip using the bar. Black arrow points on direction of insertion; **(D)** Pre-forming of the insert to the shape of 2 ml centrifugal test-tube by pressing on the bar. Black arrow indicates the direction of pressing; **(E)** The insert, removed from centrifugal test-tube after pre-forming; **(F)** Slight spreading of the sides of the insert by tweezers. Blue arrow point on direction of spreading; **(G)** Ready to use pre-formed inserts; **(H)** Magnified image of perforate zone of the insert.

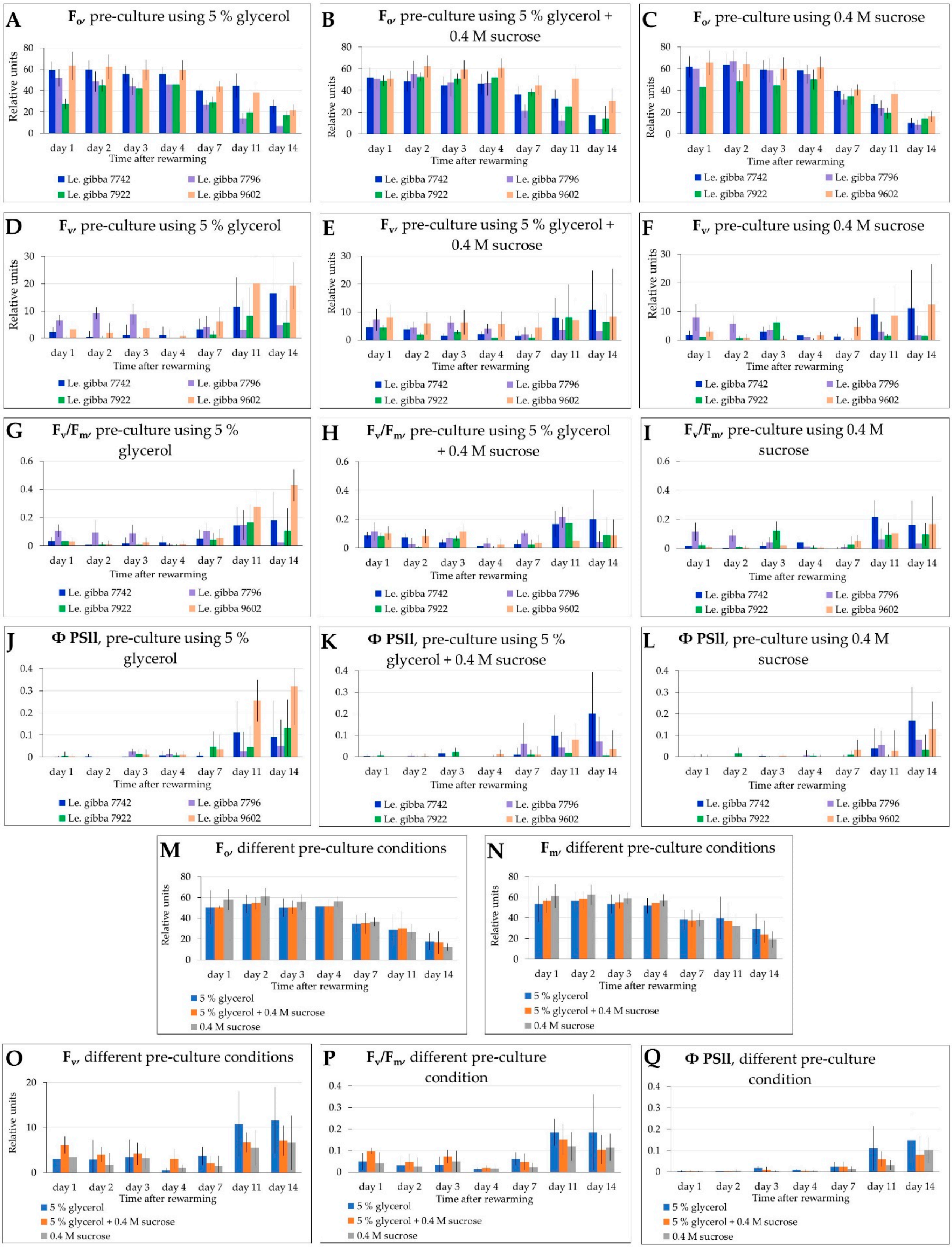

Figure S7. Dynamics of chlorophyll fluorescence parameters and photosynthetic performance of rewarmed fronds at early regrowth stage. **(A)** The minimal fluorescence level F_0 after pre-culture using 5% glycerol; **(B)** The minimal fluorescence level F_0 after pre-culture using 5% glycerol and 0.4 M sucrose; **(C)** The minimal fluorescence level F_0 after pre-culture using 0.4 M sucrose; **(D)** The maximal fluorescence level F_m after pre-culture using 5% glycerol; **(E)** The maximal fluorescence level F_m after pre-culture using 5% glycerol and 0.4 M sucrose; **(F)** The maximal fluorescence level F_m after pre-culture using 0.4 M sucrose; **(G)** The maximum quantum yield of PSII F_v/F_m after pre-culture using 5% glycerol; **(H)** The maximum quantum yield of PSII F_v/F_m after pre-culture using 5% glycerol and 0.4 M sucrose; **(I)** The maximum quantum yield of PSII F_v/F_m after pre-culture using 0.4 M sucrose; **(J)** The operating efficiency of PSII (ΦPSII) after pre-culture using 5% glycerol; **(K)** The operating efficiency of PSII ΦPSII after pre-culture using 5% glycerol and 0.4 M sucrose; **(L)** The operating efficiency of PSII ΦPSII after pre-culture using 0.4 M sucrose; **(M-Q)** Average the minimal fluorescence level F_0 **(M)**, the maximal fluorescence level F_m **(N)**, the variable fluorescence F_v **(O)**, the maximum quantum yield of PSII F_v/F_m **(P)**, and the operating efficiency of PSII ΦPSII **(Q)** for four accession of *Le. gibba* (7742, 7796, 7922, 9602).

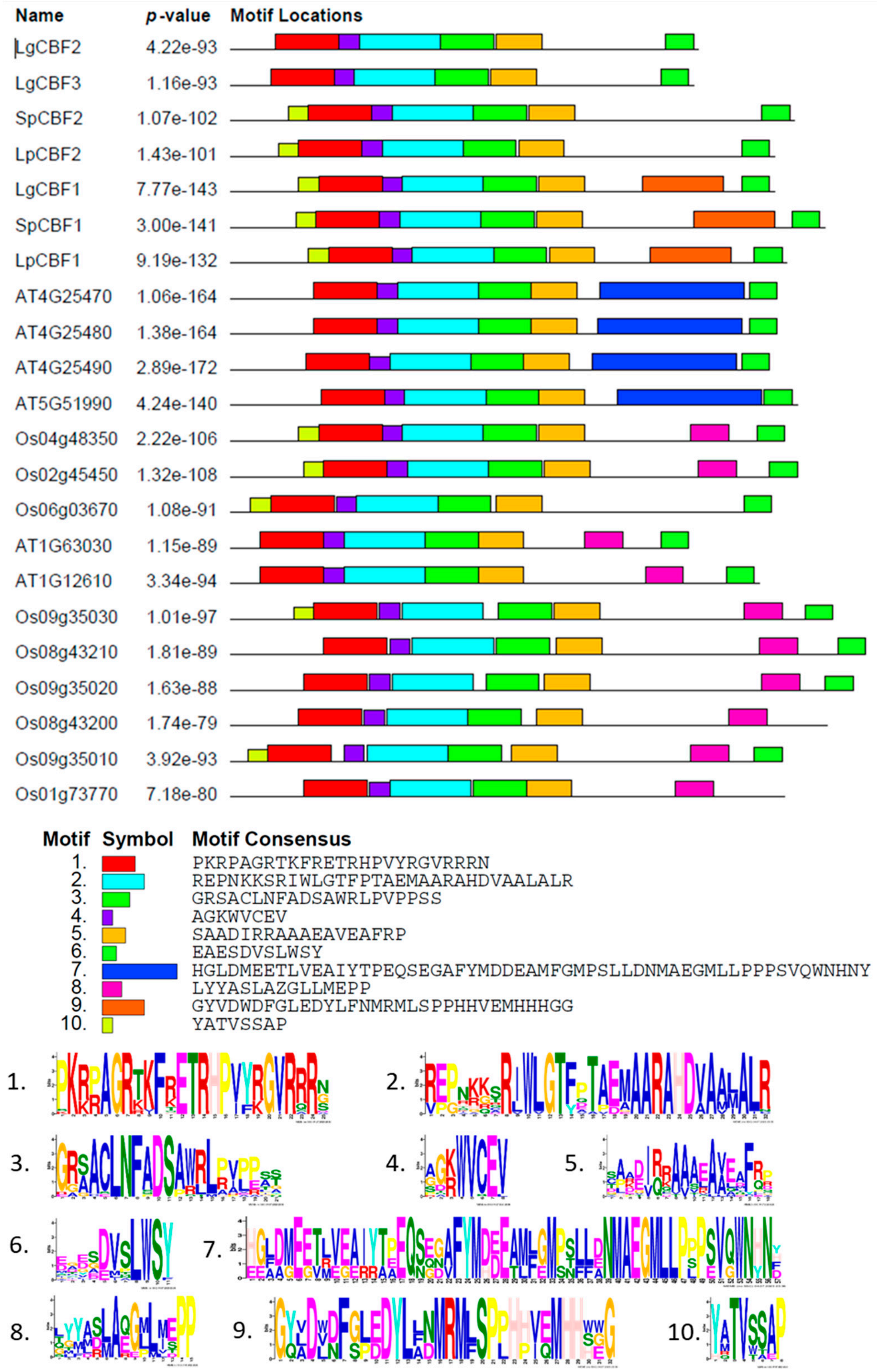

Figure S8. Structure of identified CBF/DREB1 proteins in *Le. gibba, La. punctata* and *Sp. polyrhiza* compared with homologues from Arabidopsis and rice. The 1st-5th motifs correspond to the AP2/ERF domain.

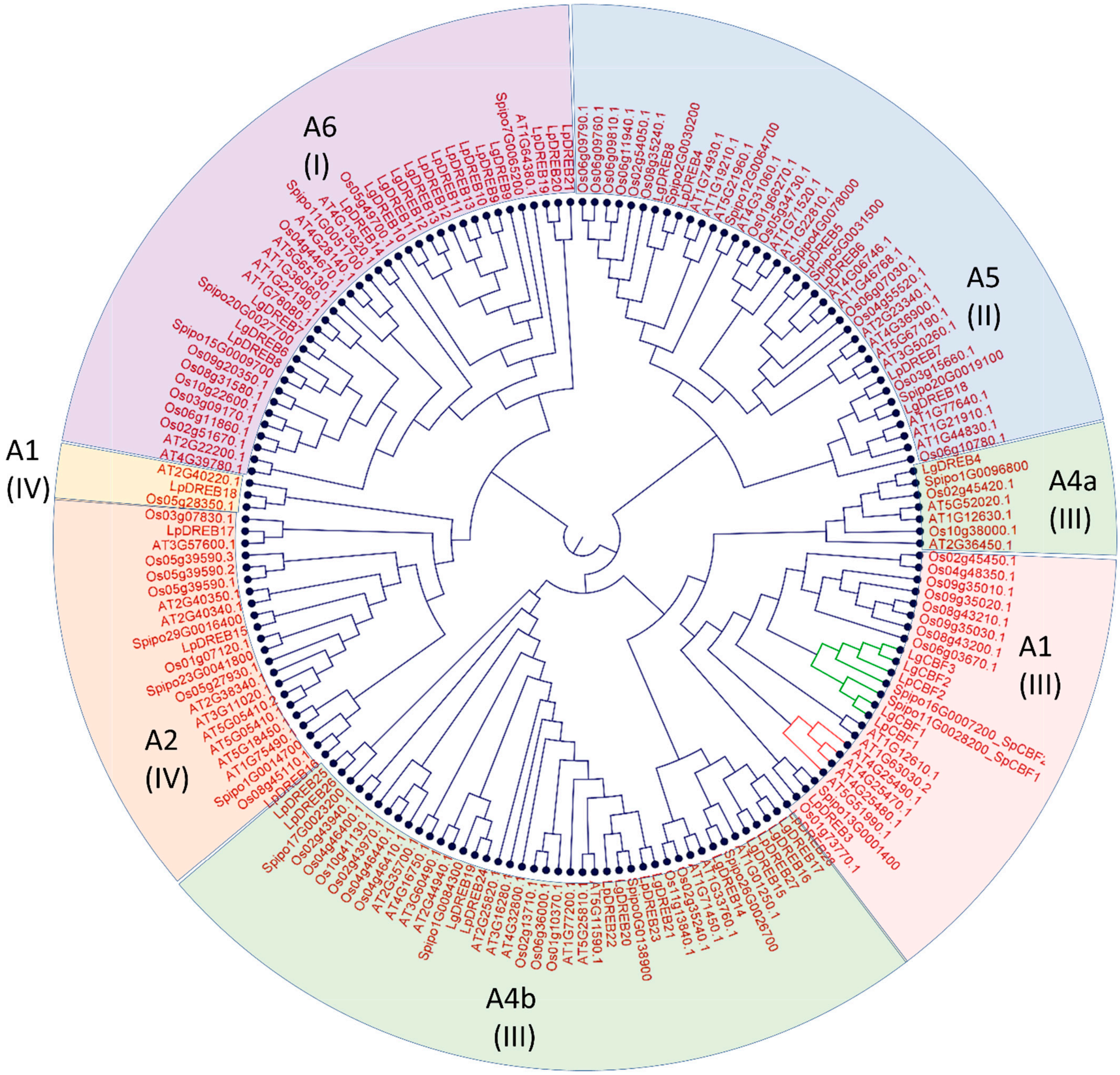

Figure S9. Phylogenetic tree of DREB protein subfamily members from Arabidopsis, rice, *Le. gibba, La. punctata* and *Sp. polyrhiza.* Green lines correspond to the duckweed *CBF* sub-tree; red lines correspond to the Arabidopsis *CBF* sub-tree., A1-A6 sub-groups of DREB were marked according nomenclature proposed in [58,59].

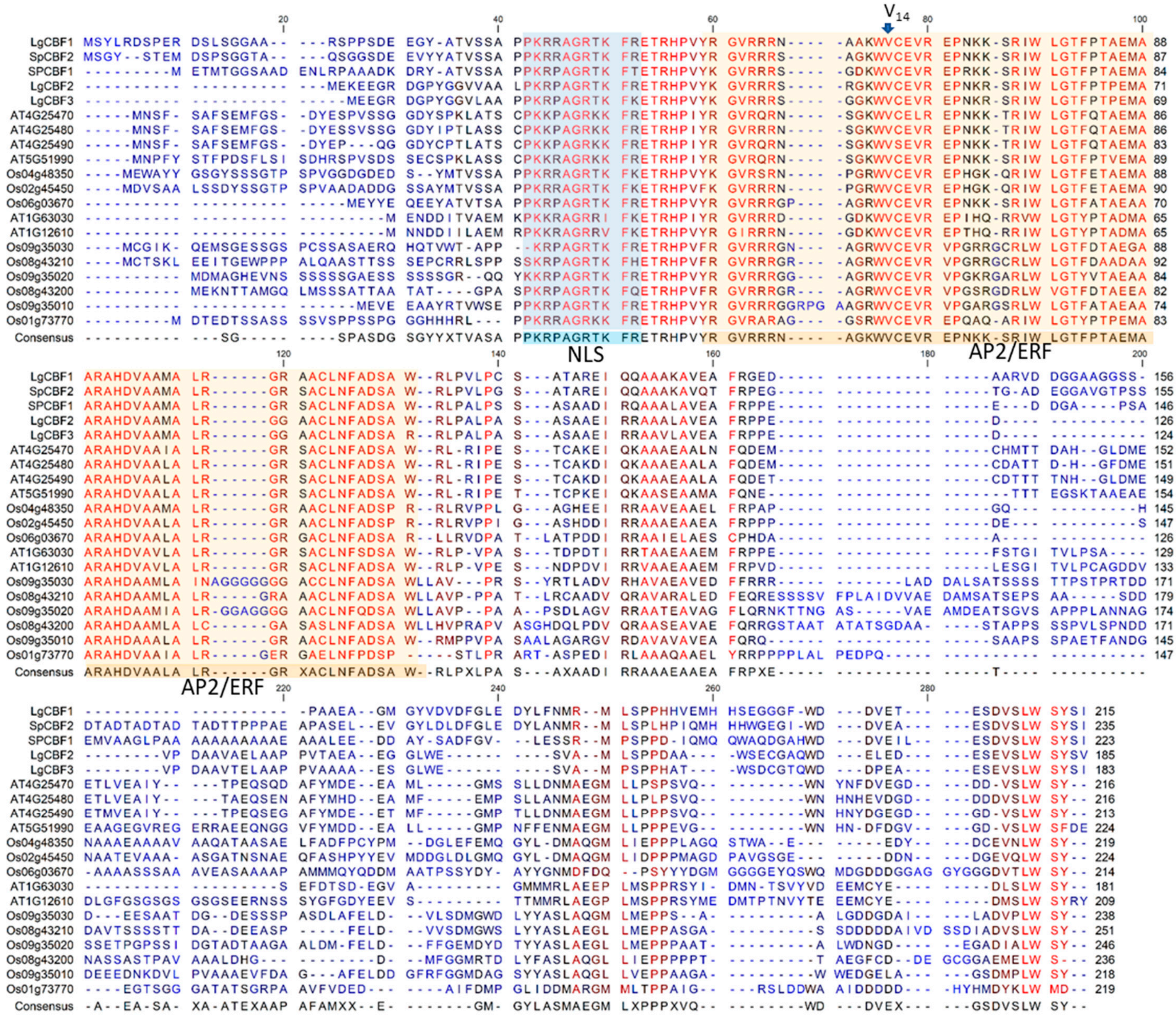

Figure S10. Sequence alignment of CBF/DREB1 proteins from Arabidopsis, rice, *Le. gibba, La. punctata* and *Sp. polyrhiza.* Nuclear localization signal (NLS) and AP2/ERF domain are highlighted with blue and orange color, respectively. Valine residue in the 14th position of AP2/ERF domain tagged with an arrow.

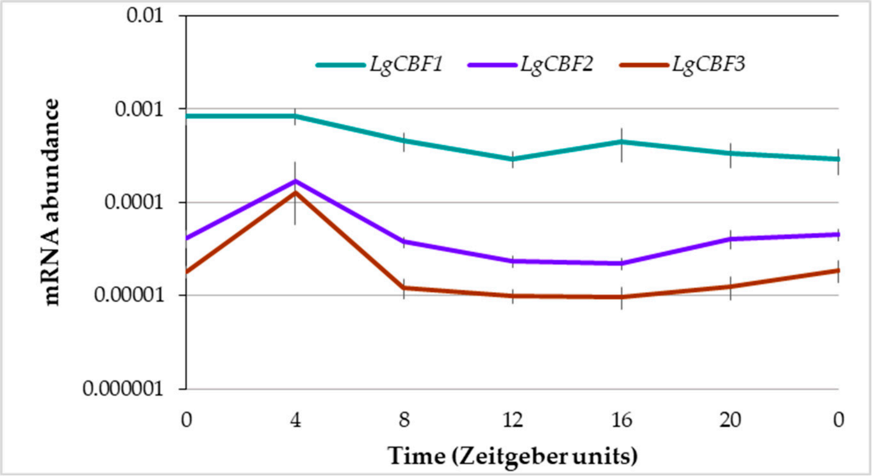

Figure S11. Relative mRNA abundance of *Le. gibba* 7742 *LgCBF1*, *LgCBF2* and *LgCBF3* during day time, relative to *histone H3* (*LgH3*). 0 corresponding to Zeitgeber unit, when the lighting in the phytochamber started. Error bars indicate standard deviations (n = 3).

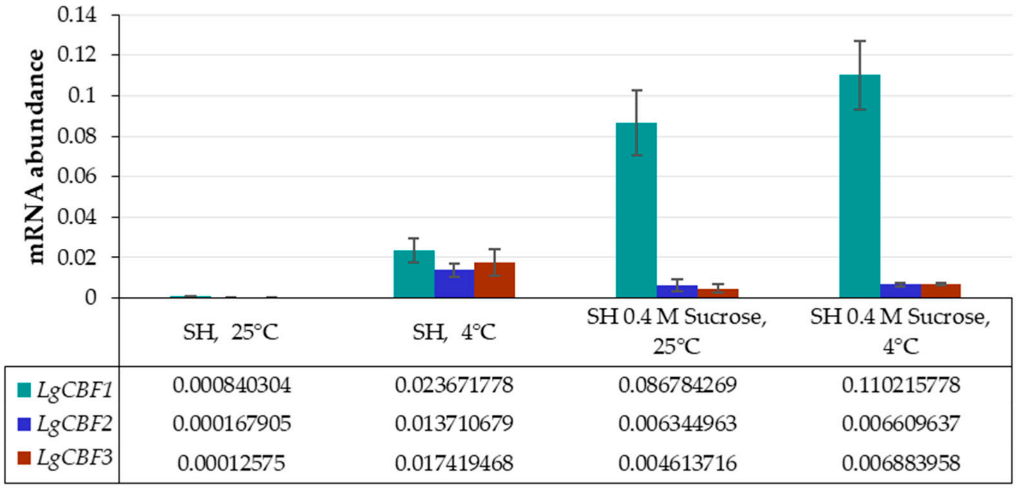

	SH, 25°C	SH, 4°C	SH 0.4 M Sucrose, 25°C	SH 0.4 M Sucrose, 4°C
LgCBF1	0.000840304	0.023671778	0.086784269	0.110215778
LgCBF2	0.000167905	0.013710679	0.006344963	0.006609637
LgCBF3	0.00012575	0.017419468	0.004613716	0.006883958

Figure S12. Relative mRNA abundance of *Le. gibba* 7742 *LgCBF1*, *LgCBF2* and *LgCBF3* genes dependent on cold and osmotic treatment, normalized against mRNA abundance of *histone H3* (*LgH3*). Error bars indicate standard deviations (n = 3).

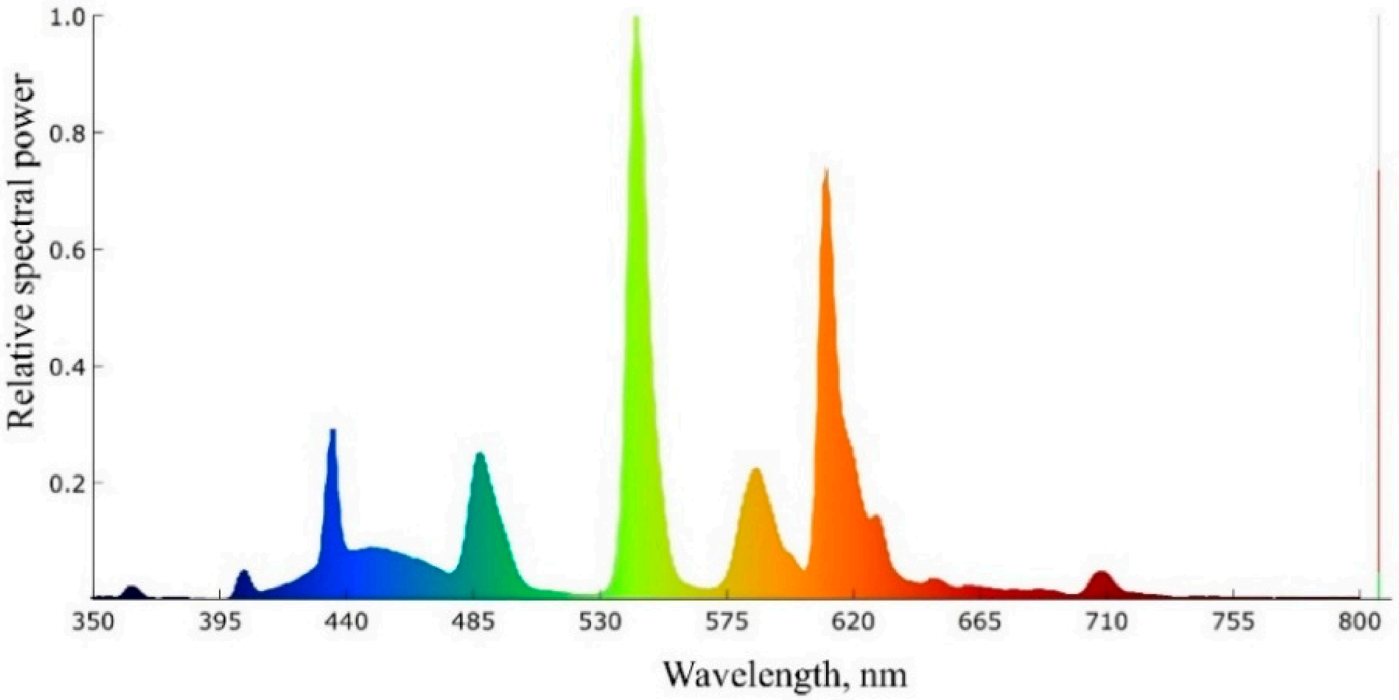

Figure S13. Relative spectral power distribution of light from light sources in the phytochamber, used in the experiments.
