# Supplementary material for: Cryopreservation of Duckweed Genetic Diversity as Model for Long-Term Preservation of Aquatic Flowering Plants
Source: Plants (Basel). 2023 Sep 18;12(18):3302. doi: 10.3390/plants12183302 (PMC10534739; doi:10.3390/plants12183302)
Supplement: Supplementary file 1 [file plants-12-03302-s001.zip › Peterson_et_al_Cryopreservation_of_duckweed_Suppl_Tables.pdf]

Table S1. Effect of supplemented compounds used in different solutions on cryopreservation results of *Le. gibba* 7742 after rewarming.

	Tested substances	Pre-cultivation	Cryoprotection (PVS3)	Washing	Re-growth	Effect on bleaching delay after rewarming
**1**	1 mM ATP	-	-	-	+	No delay, getting brown during 1-2 days
**2**	0.1 g/l casein hydrolysate	+	+	+	+	No delay, getting brown during 2-3 days
**3**	0.5 g/l glutamine,<br>0.5 g/l cysteine,<br>0.5 g/l proline	-	-	-	+	No delay, getting brown during 2-3 days
**4**	0.5 g/l PVP10000	-	-	+	+	Up to 17-20 days (in combination with attenuated illumination regime and working on ice in protected against light condition)
**5**	1 mM dithiothreitol	-	+	+	+	Up to 30 days (in combination with attenuated illumination regime and working on ice in protected against light condition)
**6**	3 mM $Na_2S_2O_3$	-	+	+	+	No delay
**7**	30 μM $Ag_2S_2O_3$	-	-	+	+	No delay
**8**	10 mM glutathione	-	-	+	+	No delay, getting brown during 2-3 days
**9**	0.4 mg/l gibberellic acid	-	-	-	+	No delay
**10**	1 mg/l 6-benzylaminopurine + 0.3 mg/l indole-3-acetic acid + 0.4 mg/l gibberellic acid	-	-	-	+	No delay
**11**	1 mg/l 6-benzylaminopurine + 0.3 mg/l indole-3-acetic acid + 0.4 mg/l gibberellic acid + 30 μM $Ag_2S_2O_3$	-	-	-	+	No delay

Table S2. Regrowth of *Le. gibba* 7742 depends on pre-culture condition.

	**Medium**	**Condition**	**Number of regrowth events after cryo, per cryo-tube** (25-50 fronds in cryo-tube)
**1**	SH, 5 g/l (15 mM) sucrose	Standard growth condition: in climate chamber (12/12h light/dark cycle, PPFD 60 $\mu mol \cdot m^{-2} \cdot s^{-1}$, 25⁰C/22⁰C for the light/dark periods)	0 - 1
**2**	SH, 5 g/l (15 mM) sucrose	Cold: in fridge (4⁰C, darkness)	0
**3**	SH, 5 g/l (15 mM) sucrose	Imitation of night frost: in climate chamber (16/8h light/dark cycle, PPFD 60 $\mu mol \cdot m^{-2} \cdot s^{-1}$, 25⁰C/-1⁰C for the light/dark periods)	0 - 1
**4**	SH, 0.4 M sucrose	Standard growth condition: in climate chamber (12/12h light/dark cycle, PPFD 60 $\mu mol \cdot m^{-2} \cdot s^{-1}$, 25⁰C/22⁰C for the light/dark periods)	2 - 4
**5**	SH, 0.4 M sucrose	Cold: in fridge (4⁰C, darkness)	0 0
**6**	SH, 0.4 M sucrose	Imitation of night frost: in climate chamber (16/8h light/dark cycle, PPFD 60 $\mu mol \cdot m^{-2} \cdot s^{-1}$,25⁰C/-1⁰C for the light/dark periods)	0 - 1

SH – nutrient medium Schenk and Hildebrand, PPFD - the photosynthetic photon flux density.

Table S3. Parameters of chlorophyll fluorescence and photosynthesis performance for intact *Le. gibbba* 7742 culture.

Parameter	F_0	F_m	F_v	F_v/F_m	Φ_{PSII}
Value	92.7±15.8	441.5±86.2	348.9±71.4	0.8±0.05	0.7±0.05

Table S4. Distribution of potential regulatory DNA cis-elements in promoter region of *CBF* genes in *A. thaliana* and duckweed.

cis-element	*AtCBF1*	*AtCBF2*	*AtCBF3*	*LgCBF1*	*LgCBF2*	*LgCBF3*	*LpCBF1*	*LpCBF2*	*SpCBF1*	*SpCBF2*
G-box	2	1	2	3	1	2	1	5	2	2
E-box	6	5	10	6	9	18	8	11	10	11
EE, evening element*	2	3	3	0	0	0	0	0	0	0
CGCG-box, CAMTA binding site**	1	2	3	7	4	5	12	4	4	4
CBS, CCA1 binding site*	7	6	7	3	5	4	1	3	1	2
MYB*	6	5	7	1	2	0	3	4	2	9
BRRE, brassinosteroid response element	3	0	0	0	0	1	7	5	9	1
T1ME, TOC1 morning element*	21	13	24	4	7	14	11	16	9	7
ABRE, abscisic acid responsiveness	4	2	7	4	4	6	6	5	15	6
MeJA-responsiveness**	3	0	2	5	3	4	11	3	10	4
ERE, Ethylene-responsive element**	1	0	1	4	1	3	1	0	0	0
MYC, Water stress and dehydration responsiveness	1	3	7	2	2	10	1	1	0	3
TCA-element, salicylic acid responsiveness*	1	2	1	0	1	0	2	2	1	0
TGA-element, auxin-responsive element	0	1	1	1	0	1	1	1	0	2

Reduced (*) and increased (**) number of *cis*-elements compared to *Arabidopsis* are marked in blue and red, respectively.

Table S5. List of RT-qPCR primers used for evaluating expression of the *CBF* genes in *Le. gibba, La. punctata* and *Sp. polyrhiza*.

Gene	Forward primer	Reverse primer	PCR product, length in bp
LgCBF1	CTCTCCGCCTCATCACGTC	GCAAGGATATGCCGCGTC	137
LgCBF2	TCCGTCTGAACTAAGAGGCG	ACACATAGTAGCTCCCAACGG	100
LgCBF3	CGATAGTGGAGAGGTGCCA	CAGCCATGCCGTTGTATTGA	102
LgHistone3	CCGCAAGTACCAGAAGAGCA	AGGTATGCTTCGGCTGCTTC	147
LgActin	TGAGCATGGAATCGTCAGCA	GCCTTCGGATTCAGAGGAGC	129
LpCBF1	TCCAAGGAACTCTCGGCCAT	GTGGCGTAAGTCTCCTCGTC	119
LpCBF2	TTGGGACTACTGGAGAGCGT	GGGACACGTCAGACTCTGTG	103
LpHistone3	CCCTCCGTGAAATTCGCAAG	GGCTCTGGAACCTCAGATCG	147
SpCBF1	ACCTGGCCAACATGAGGATG	CGCCACGTCAGATGGAGTAG	132
SpCBF2	GAGAGCTCGAGGATGCCTTC	CAGAGGGACACGTCGGATTC	107
SpHistone3	CGCAAGTACCAGAAGAGCAC	CGAAGAGACCCACGAGGTAG	160
SpActin	TGTTTTCCCAAGTATCGTC	TCCCAGTTGGTGACGATT	153
